# Attribute Embedding: Learning Hierarchical Representations of Product Attributes from Consumer Reviews

**DOI:** 10.1177/00222429211047822

**Published:** 2021-11-17

**Authors:** Xin (Shane) Wang, Jiaxiu He, David J. Curry, Jun Hyun (Joseph) Ryoo

**Keywords:** attribute hierarchy, attribute embedding, machine learning, word2vec, meta-attribute, natural language processing

## Abstract

Sales, product design, and engineering teams benefit immensely from better understanding customer perspectives. How do customers combine a product's technical specifications (i.e., engineered attributes) to form abstract product benefits (i.e., meta-attributes)? To address this question, the authors use machine learning and natural language processing to develop a methodological framework that extracts a hierarchy of product attributes based on contextual information of how attributes are expressed in consumer reviews. The attribute hierarchy reveals linkages between engineered attributes and meta-attributes within a product category, enabling flexible sentiment analysis that can identify how consumers receive meta-attributes, and which engineered attributes are main drivers. The framework can guide managers to monitor only portions of review content that are relevant to specific attributes of interest. Moreover, managers can compare products within and between brands, where different names and attribute combinations are often associated with similar benefits. The authors apply the framework to the tablet computer category to generate dashboards and perceptual maps and provide validations of the attribute hierarchy using both primary and secondary data. Resultant insights allow the exploration of substantive questions, such as how Apple improved successive generations of iPads and why Hewlett-Packard and Toshiba discontinued their tablet product lines.

Product attributes have long played a central role in marketing, particularly in competitive positioning, brand strategy, and new product development. However, the product attribute as a construct remains slippery, given that a product can be characterized in dozens of ways from multiple perspectives. These characterizations include basic physical characteristics (e.g., weight, length, chemical composition), intangible properties (e.g., country of origin, price, brand name), and increasingly abstract ideas (e.g., product quality, brand equity, ethicality). The difficulty in conceptualizing attributes was apparent decades ago when Howard and Sheth (1969) first proposed a multilevel hierarchy that distinguished concrete attributes from more abstract attributes and Lancaster (1966, p. 134) clarified that a “good, per se, does not give utility to the consumer” but “possesses characteristics, and these characteristics give rise to utility.”

The increasing applications of marketing methods based on product attributes, such as conjoint analysis and market structure analysis (MSA), intensified the need for a method that could properly identify the attributes as perceived by consumers (Hauser and Clausing 1988; Luce and Tukey 1964). However, the vast majority of marketing studies have focused on concrete and technical attributes extracted from lists of ingredients or technical specification sheets (Gustafsson, Herrmann, and Huber 2007). Although these features are easier to identify, they may be irrelevant to the product benefits that impact consumer evaluations. Accordingly, the current research directly uses the “voice of consumers” (Berger et al. 2020) to find the most relevant drivers of consumer needs and uncover the relationships between easily identifiable attributes and abstract product benefits that truly matter for consumers’ preferences and choices (Kim et al. 2017). We define a product's technical specifications, which are easily identifiable, as “engineered attributes,” and its perceived benefits that fulfill consumers’ needs as “meta-attributes” (Netzer et al. 2008).

Understanding how concrete product attributes form higher-level benefits for consumers can benefit various corporate teams. For example, sales teams need to understand the high-level product benefits that drive consumer buying behavior. Product design teams must communicate with engineering and manufacturing to understand the relationships between the product's technical specifications and its perceived benefits. Engineering teams are heavily impacted by design changes, and they need to be able to estimate the trade-offs of technical subcomponents to build the product model that fulfills the more abstract benefits associated with the product's meta-attributes. The traditional method of surveys can be time consuming and may yield static results that are inconsistent across different sampling periods. Thus, there remains a significant gap in the literature: How can the link between engineered attributes and meta-attributes be uncovered directly from consumer input to inform managerial decisions?

To fill this gap, we devise a methodological framework that we name the “attribute embedding model,” which is based on machine learning and natural language processing (NLP) to obtain an embedded representation of product attributes. Specifically, “embedded representation” describes (represents) textual data such as individual product attributes using the words that surround such textual data (i.e., the contextual information) in consumer reviews. The representation is quantified using a neural network that enables us to mathematically measure the degrees of similarity between various product attributes based on how they are described by consumers themselves (i.e., the contextual information), revealing similarities and differences in the attributes’ usage by consumers. From this embedded representation, our model then identifies multilevel clusters of product attributes that reflect the levels of abstract product benefits. Furthermore, we can use the sentiments associated with these meta-attributes to evaluate objects of managerial interest, such as a product or brand, and then drill down to examine which engineered attributes primarily drive consumer sentiments in relation to the meta-attributes.

Our research makes three main contributions. First, we provide a methodological framework for managers to monitor and extract information related to products and their attributes from consumer reviews based on the context. Confronted with unstructured free-form reviews, managers may struggle to determine how to navigate and monitor only those portions of review content that are relevant to specific attributes of interest. Moreover, comparing products within brands (e.g., iPad 1 and iPad 2) and between brands (e.g., Apple and Samsung) can pose difficulties because of inconsistent names and combinations of engineered attributes that provide similar benefits. Because our framework exploits the contexts surrounding product attributes expressed in consumer reviews, managers can use it to directly monitor how meta-attributes evolve within brands and to compare brands within a product category to inform their product-related decisions. Using primary and secondary data, we provide validations that our hierarchical structure of meta-attributes adequately approximates consumers’ underlying review-writing behaviors. We also show that our hierarchy is predictive of real-world performance metrics.

Second, we extend sentiment analysis of consumer reviews by demonstrating hierarchical sentiment analysis, which aggregates sentiment scores associated with individual attributes based on our attribute hierarchy. Starting at the review level, sentiment scores can be aggregated upward to yield insights for various units of analysis, such as stockkeeping unit (SKU), product series, and brands. Using hierarchical sentiment analysis, managers can go beyond relying on review ratings, which only describe products as a whole and cannot be accredited to specific product attributes. We demonstrate that this flexible approach to sentiment analysis can generate tailored dashboards and perceptual maps from consumer reviews that can help inform managerial decisions. We also find that hierarchical sentiment analysis leads to the greatest improvement in forecasting sales compared with simpler approaches to sentiment analysis.

Third, we use consumer reviews of tablets to provide not only a practical demonstration of our method, but also substantive contributions for the tablet product category. In particular, we analyze consumer sentiments about Hewlett-Packard (HP) and Toshiba to explore potential reasons why these two brands ultimately discontinued their tablet product lines. Using our attribute hierarchy, we evaluate their meta-attributes and then drill down to the level of engineered attributes to find that the limited number of apps available for HP's tablets and the thickness and weight of Toshiba's tablets were the main drivers of consumers’ negative sentiments about the products. We then analyze the meta-attributes of market-leading brands Samsung and Apple, as well as a product series from each firm in the same time period, to explore potential drivers of their successes. Berger et al. (2020, p. 1) note that “for data to be useful, researchers must be able to extract underlying insight—to measure, track, understand, and interpret the causes and consequences of market behavior.” In this sense, our method is highly useful for developing marketing strategies, as it provides valuable insights on the relationships between product attributes and consumer valuations, thereby improving firm performance.

## Related Literature

### Theories of Attribute Hierarchy

The idea that product attributes range from concrete to abstract has a long history in marketing (Johnson 1988, 1989; Johnson et al. 1992) and is grounded in cognitive theory, relational learning theory, means–end theory, and consumer decision theory. Ultimately, a consumer choosing from a product assortment asks, “Will product x be more valuable to me than product y?” More complex products, such as tablet computers, require numerous apples-to-oranges comparisons because they differ on many engineered attributes. For instance, what combination of engineered attributes (e.g., antiglare glass, display size, in-plane switching, intelligent color display) results in a desirable tablet display? Cognitive theory stresses the limitations of human memory and information processing capacity—constraints that complicate choices for consumers confronted with multiple attributes (Payne, Bettman, and Johnson 1993). Compounding the problem, product choices require inferences based on not only individual attributes but also relations between attributes (Bettman, Luce, and Payne 1998).

Given these constraints, relational learning theory shows that consumers implicitly group engineered attributes to form higher-level attributes that help them with multidimensional comparisons. For example, information processing for consumers can become less challenging if engineered attributes such as screen resolution (e.g., pixels) and backlighting methods (e.g., RGB LEDs, cathode fluorescent) are grouped in the same higher-level attribute, “visual clarity” (Kibbe and Feigenson 2014). This process ultimately reduces the number of engineered attributes to satisfy cognitive limitations and crystallizes the differences between products on higher-level dimensions (Chen, Lu, and Holyoak 2015).

These theories indicate that attribute hierarchies form naturally as consumers search within a product category. Evidently, the relationships between product attributes are often expressed in consumer reviews. For example, consider the following texts from the same review: “With the Bluetooth feature you can stream music to speakers,” and “I recently acquired a pressure sensitive stylus that connects via Bluetooth.” For this particular customer, we can infer that Bluetooth is an attribute that connects hardware devices to a central processor. In a separate review, we find, “The USB supports keyboards, flash drives/external hard drives formatted under FAT.” We can therefore infer that USB, which is a physical attribute, unlike Bluetooth, satisfies a need in common with Bluetooth—connectivity. The managerial value in understanding how individual attributes, such as Bluetooth and USB, individually load onto meta-attributes, such as connectivity, becomes clear*.* We devise a methodological framework that mines customer reviews to extract patterns of contextual information surrounding engineered attributes, revealing which attributes satisfy similar “needs, motivations, and goals” of consumers (Netzer et al. 2008, p. 345).

### Text Mining and User-Generated Content

Prior research suggests that user-generated content (UGC) contains information regarding customer needs comparable to that collected by market research firms (Timoshenko and Hauser 2019). Given the wide availability of UGC, scholars have focused their methodological efforts on using it as a source of consumer insights. In this research domain, machine learning and NLP have been applied because they are well-suited to quantify information from unstructured data. The application of machine learning to UGC has not only led to the development of new methods but also contributed insights related to the underlying behaviors of consumers. For example, Liu, Lee, and Srinivasan (2019) extract content related to quality and price from customer reviews and quantify their causal impact on sales while accounting for users’ review-reading behaviors. Melumad, Inman, and Pham (2019) analyze the linguistic characteristics of online reviews and find that because reviews written on smartphones tend to be more restricted and shorter, consumers express more emotion when writing reviews on smartphones than when they write reviews on personal computers. Netzer, Lemaire, and Herzenstein (2019) analyze the texts of loan requests on a peer-to-peer lending platform and find that textual information contains traces of psychological differences between borrowers that improve the prediction of loan defaults.

Our focus on UGC and product attributes places our research close to the methodological domain of MSA. However, our research differs from previous efforts in this area in that our main objective is to uncover the relationships between product attributes and how they form abstract benefits for consumers, whereas the main objective of MSA is to summarize the valence of product attributes to a small number of dimensions that can then be visualized on a perceptual map. Nonetheless, given the intermediary step in MSA that extracts product attributes from UGC, we review the literature in this domain and highlight differences with our work, as summarized in Table 1.

**Table 1.** Comparisons to Published Research in MSA Using Text Mining.

	**Traditional Methods**	**Lee and Bradlow (2011)**	**Netzer et al. (2012)**	**Tirunillai and Tellis (2014)**	**Moon and Kamakura (2017)**	**This Article**
**Data Acquisition**						
Source:	Surveys	Pro/con product reviews	Free-form web forum	Free-form online reviews	Free-form expert reviews	Free-form online reviews
Cost:	High	Low	Low	Low	Moderate^a^	Low
**Analysis Methods**						
Similarity between brands:	Judged	Attribute counts	Comentions of brands	LDA: dimension heterogeneity	Comentioned topics	Sentiment for product attributes
Dimensionality reduction:	No	Review × word count matrix	Co-occurrence matrix	Dirichlet posterior	Review × topic frequency matrix	Embedded representation
Linguistic structures and local context:	Yes (manually)	No	No	No	No	Yes (data-driven)
Consumer sentiment:	Yes	Via human labeled pos/neg	Ad hoc analysis: common problems^b^	Yes	Yes, by topic (deduced from overall rating for wine)	Sentiment analysis via machine learning
**Implementation and Results**						
Software availability:	Proprietary	Unpublished code	Commercial software (e.g., SPSS text analytics)	Proprietary software (patent pending)	Open source and proprietary model	Open source
Attribute structure:	Yes, varies by study	Single level	No	No	Hierarchical structure (topics; judged manually)	Hierarchical structure (attributes; data-driven)
Product usage:	Surveys	No	Ad hoc analysis: common problems^b^	No		Embedded representations; semantic vectors
Validation data:	Varies by study	Survey data and *Consumer Reports* magazine	Transaction data	Survey data and *Consumer Reports* magazine	Split-half (wine data); predictive likelihood vs. Lee and Bradlow (2011)	Survey data, *Consumer Reports* magazine, Epinions, Amazon, eBay, and news proxies for transaction data

^a^ Method relies exclusively on expert reviews (vs. user reviews). The method is costly to scale because each domain of application requires new topics organized via human judgment.

^b^ Authors did ad hoc analysis to find the common problems mentioned in reviews using a function available in SPSS’s Text Analytics procedure. *Notes*: MSA = market structure analysis; LDA = latent Dirichlet allocation.

The early methods for MSA are based on word frequency. Lee and Bradlow (2011) extract product-related words and phrases from semistructured positive or negative reviews on Epinions.com to construct a review × word count matrix. Similarly, Netzer et al. (2012) extract product-related words from forum messages to construct a symmetric word × word co-occurrence matrix. These matrices are then used with correspondence analysis and multidimensional scaling to generate product positioning maps. However, matrices based on frequency are often very sparse due to consumers’ use of unique and rare words. Consequently, consumer perceptions for only the most frequently mentioned attributes can be clearly interpreted, whereas less frequently mentioned attributes are either dropped from the data during cleaning or potentially produce biased results driven by a vocal minority of consumers. The matrices also implicitly assume that each word or product attribute is independent of another. As a result, rich information can be lost regarding the similarities and differences of product attributes and how attributes are used interchangeably or complemented in certain contexts.

Tirunillai and Tellis (2014) extend the frequency-based approach using the latent Dirichlet allocation (LDA) approach. Their method generates a posterior distribution of topics uncovered from the texts of customer reviews, resulting in a small fixed number of topics that summarizes the entire review corpus. This review × topic matrix addresses the sparsity problem of frequency-based approaches; rare and unique attributes can be assigned to topics that contain more frequently mentioned attributes, which makes it easier to interpret them with respect to general consumer perceptions. However, the bag-of-words assumption inherent to LDA implies that the topics in the matrix are treated as independent, overlooking information related to attribute relationships in a similar way to frequency-based approaches. Moreover, the LDA approach measures the valence of topics, rather than the specific product attributes within the topics. The valence of product attributes within a single topic can then be interpreted only as a whole and cannot be linked to specific SKUs, or to product series within brands, that may vary in valence.

To address these drawbacks, Moon and Kamakura (2017) develop ontology-learning-based text mining, which uses human expertise to construct a hierarchical taxonomy consisting of grand topics, subtopics, and terms from consumer reviews. The authors apply this method to the wine and hotel industries. The experiential nature of these industries means that they benefit from the hierarchical structure because it captures the relationships between multiple topics that form complex consumer experiences. Although this hierarchical taxonomy resembles our attribute hierarchy, the focus of the taxonomy is not on uncovering relationships between product attributes but rather on general themes within experiential industries deemed important by human experts. Consequently, the insightfulness of the taxonomy is constrained by human judgment and can be expensive to construct periodically as different managerial goals necessitate the development of completely new taxonomies (with different topics and subtopics).

Our work is methodologically similar to that of Timoshenko and Hauser (2019), who identify customer needs in reviews of oral care products. As in our research, the authors distinguish between lower-order attributes and higher-order needs, defining the latter as “an abstract context-dependent statement describing the benefits, in the customer's own words, that the customer seeks to obtain from a product or service” (Timoshenko and Hauser 2019, p. 2; see also Griffin and Hauser 1993). In addition, Timoshenko and Hauser (2019) employ word2vec to clean the review corpus of uninformative sentences and cluster their method's output. However, their work differs from ours in that the authors rely on supervised learning using manually labeled sentences to identify those about customer needs. Further, they focus exclusively on attribute identification, which comprises only one step in our attribute hierarchy. At the same time, their work complements our research because it provides a strong basis for the use of reviews to understand consumer valuations: the content of customer reviews contains 97% of customer needs identified by a research firm using traditional surveys conducted over a 30-year period (Timoshenko and Hauser 2019).

Because the main focus of our research is to uncover the relationships between product attributes in a data-driven manner, the neural network that is part of our framework addresses sparsity concerns by projecting product attributes drawn from customer reviews onto a fixed-dimensional vector space. Whereas rare words that consumers use can pose problems of interpretability for frequency-based methods, our method can evaluate the usage contexts of rare words against other words used in similar contexts. In turn, this improves the clustering of engineered attributes into meta-attributes, the accuracy of measuring sentiment expressed toward individual attributes, and the overall informative value of our hierarchy.

## Methodological Framework

### Overview of the Attribute Embedding Model

**Figure 1.**
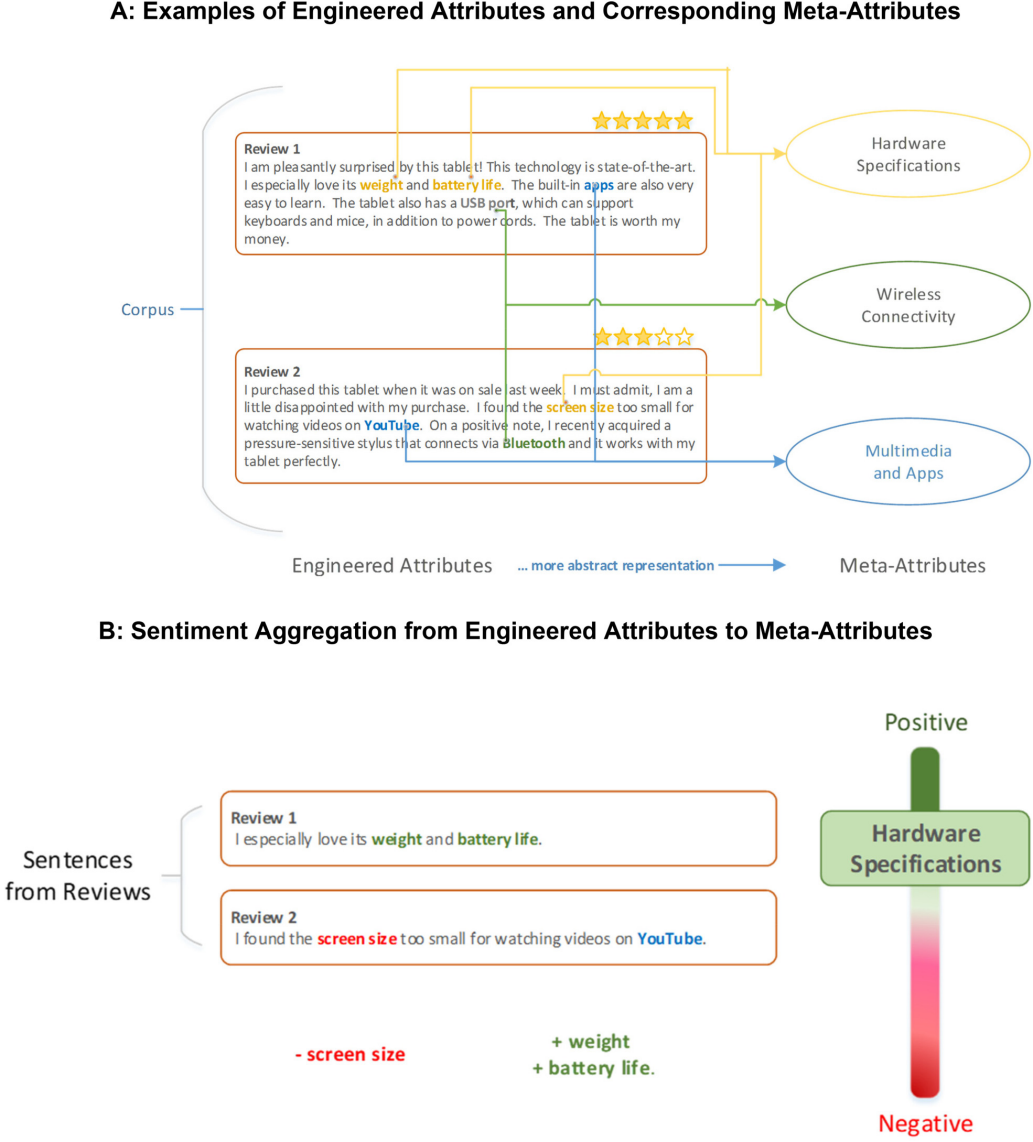
Illustration of measuring meta-attribute sentiment.

We illustrate our method using customer reviews of tablets. The tablet category is characterized by scores of product attributes that consumers evaluate differently depending on their understanding and experience with the category and on their desired benefits. For exposition, we use the running example in Figure 1, Panel A, to illustrate key terms. We begin with a corpus, which is a collection of texts. In our case, the corpus comprises consumer reviews of tablets from Amazon. After cleaning our corpus (e.g., removing commonly used but uninformative stop words such as “I,” “the,” “was,” and “a”), each word is classified according to its part of speech (e.g., noun, adjective, verb), a process known as parsing. Through parsing, we identify nouns and noun phrases in our corpus that encapsulate a product's low-level technical specifications, which we term “engineered attributes.” Examples of engineered attributes in the context of tablets include “weight,” “battery life,” “apps,” and “USB port” in Review 1, and “screen size,” “YouTube,” and “Bluetooth” in Review 2 of Figure 1, Panel A. Our objective is to identify, in a data-driven manner, the relationships between engineered attributes and high-level product benefits, which we call “meta-attributes” (Netzer et al. 2008). Consumers are unlikely to form preferences solely based on a product's technical specifications. They are more likely to consider abstract benefits, constructed using mental processes and existing product knowledge, which are reflected in an implicit (i.e., seldom-expressed, often impossible to articulate) combination of engineered attributes. For example, in Figure 1, Panel A, there are three meta-attributes: “Hardware Specifications,” “Wireless Connectivity,” and “Multimedia & Apps.” Each meta-attribute is a combination of several engineered attributes; for example, Hardware Specifications contains weight, battery life, and screen size.

We uncover meta-attributes from the extracted engineered attributes by applying word2vec (Mikolov, Chen, et al. 2013; Mikolov, Sutskever, et al. 2013). Word2vec uses a neural network that takes a corpus as its input to estimate a vector for each word in the corpus as its output. These semantic vectors exist in a vector space where words that are expressed in similar contexts are located close together (e.g., YouTube is located closer to apps than to weight). We use word2vec to obtain semantic vectors for the engineered attributes and use cosine similarities to measure the Euclidean distance between these vectors. Subsequently, we use these distances in hierarchical clustering to reveal clusters of engineered attributes that identify meta-attributes.

The engineered attributes that make up a meta-attribute can each be evaluated differently by consumers. Consider the two examples in Figure 1, Panel B. The sentence in Review 1 associated with the meta-attribute of Hardware Specifications (i.e., “I especially love its weight and battery life”) evaluates the two engineered attributes positively. However, the sentence in Review 2 associated with Hardware Specifications (i.e., “I found the screen size too small for watching videos on YouTube”) evaluates the engineered attribute negatively. These examples demonstrate how a meta-attribute can be composed of both positive and negative valence at the engineered-attribute level. In the next section, we outline how our method preserves the information regarding the valence of engineered attributes when aggregating the engineered attributes upward to obtain meta-attributes. We illustrate this aggregation using the example of a gauge on the right in Figure 1, Panel B, which summarizes the valence of engineered attributes contained in two customer reviews at the meta-attribute level.

### A Model for Semantic Representation

**Figure 2.**
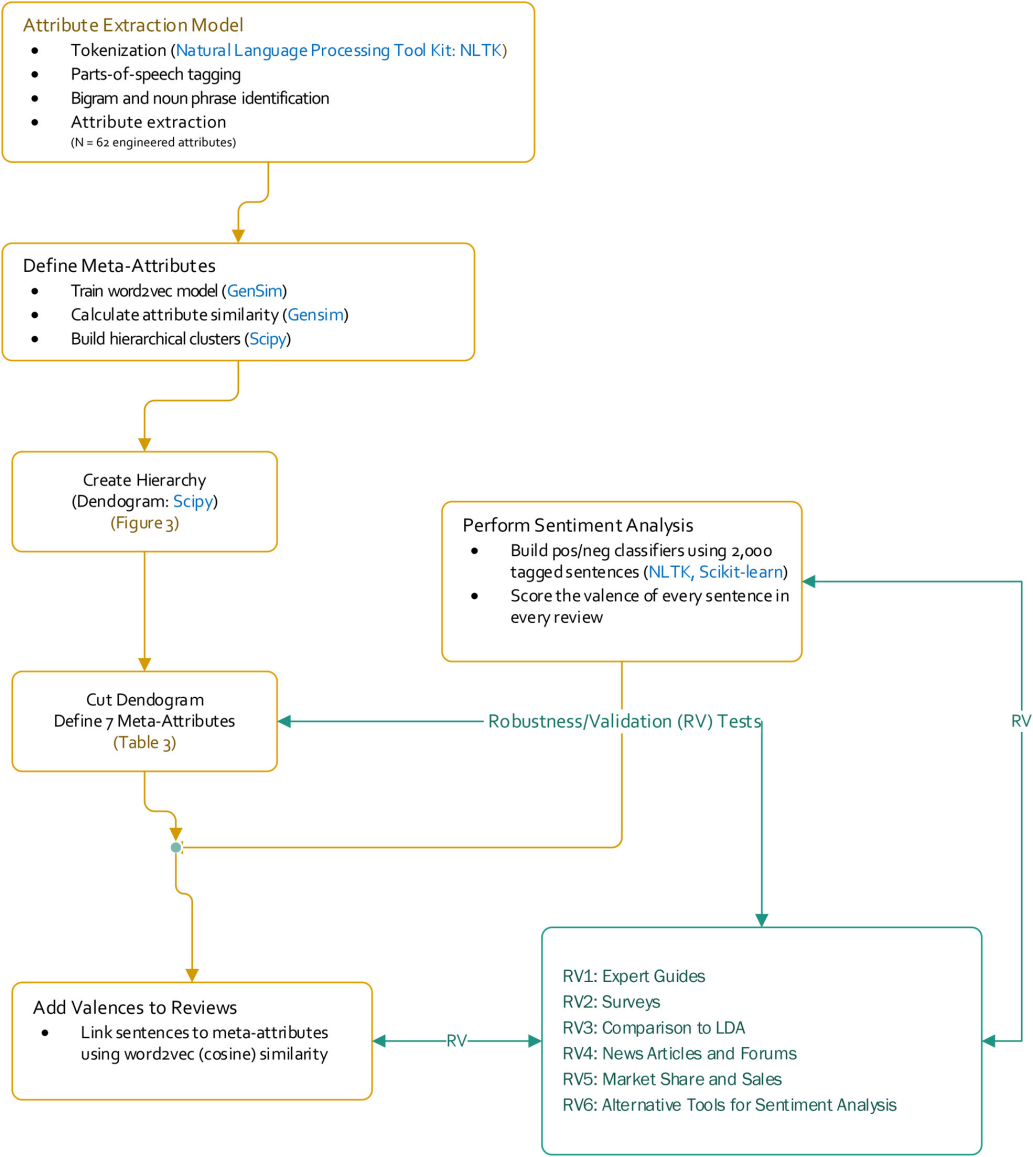
Major steps in the workflow for this research.

Traditional NLP methods can convert text into vectors, but the conversion is simply based on word counts by encoding a corpus into an n (reviews) × m (unique words in corpus) matrix. As we have noted, word count matrices are usually sparse. Word2vec, however, uses a neural network approach—the skip-gram model—to predict the context in which a given word will appear. A given word's context is simply the set of words surrounding it. Due to its predictive nature, the skip-gram model is extremely efficient at preserving as many properties of the original data as possible when moving from the corpus to the lower dimensional embedding space. Data “cleanup” is performed first to rid each review of nuisance information, such as articles and conjunctions, and to standardize the form of “words” (nouns and noun phrases that consumers use to describe product attributes). Engineered attributes are extracted at this step and fed as input into the word2vec model (see Figure 2 and pseudocode in the Appendix).

Formally, we wish to represent an engineered attribute w using a d-dimensional semantic vector v_w_. The skip-gram model aims to maximize the log probability shown in Equation 1.

(1) $$\frac{1}{\left|V\right|}\overset{\left|V\right|}{\underset{t=1}{\sum }}\;\underset{-k\leq j\leq k,j\neq 0}{\sum }\;\log\;p(w_{t+j}|w_{t}).$$

In expression Equation 1, an engineered attribute is a noun or noun phrase at location t denoted by w_t_. The log-likelihood, $\log\;p(w_{t+j}|w_{t})$ , is the probability of another word appearing in the surrounding context at location t + j. This probability is summed over j within a context window of size k (usually between 5 and 10 words) on either side of w_t_ (i.e., −k≤j≤+k ) and then summed over the entire corpus vocabulary V with size |V| . For example, consider the following sentence: “Highly durable while sensitive to user touch, the resistive screen makes display navigation and multitasking pleasant.” The engineered attribute is resistive screen, and its context is the k words that are observed before and after its location in the sentence. Assuming k = 5, the training sample for the skip-gram model, after the removal of stop words, contains {highly, durable, sensitive, user, touch, makes, display, navigation, multitasking, pleasant}. The skip-gram model maximizes the probabilities of observing each of these words near the engineered attribute at their respective locations. The goal is to estimate the model's parameter vector θ to maximize the corpus probability, as shown in Equation 2.

(2) $$\underset{\theta }{\arg\;\max}\;\underset{w\in V}{\prod }\;\underset{c\in c(w);c(w)\subseteq C}{\prod }\;p(c|w;\theta )=\underset{\theta }{\arg\;\max}\;\underset{\mathrm{All}\;(w,c)}{\sum }\;\log\;p(c|w;\theta ).$$

In Equation 2, C is the set of all available contexts and c(w) is the set of all contexts for a specific attribute w. The semantic vectors of w and every c∈c(w) are denoted v_w_ and v_c_, respectively. The parameter vector θ consists of v_w_’s in $R^{d}$ for w∈V and v_c_’s in $R^{d}$ for c∈c(w) , for a total of |C|×|V|×d individual parameters. To estimate θ, a single hidden-layer neural network first projects an attribute w to a vector v_w_ in $R^{d}$ . Then, a multinomial logit model is trained using the v_w_’s as the independent variables to predict the conditional probability of the context given the attribute as shown in Equation 3.

(3) $$p(c|w;\theta )=\frac{\exp(v_{c}^{T}v_{w})}{\sum_{c^{\prime }\in C}\;\exp(v_{c^{\prime }}^{T}v_{w})}.$$

Here, the v_c_’s can be viewed as the β’s in the usual multinomial logit model. The log-likelihood of the entire model is computed by summing over all (w,c) combinations, resulting in Equation 2. Maximizing Equation 2 as a function of θ yields the maximum likelihood estimates of the semantic vectors v_w_’s. For the summary of models and equations, see Web Appendix A.

Without capturing context, resistive screen in our previous example may be classified based on word co-occurrence to the meta-attribute Hardware Specifications, which contains the engineered attributes screen size, screen resolution, and inch screen. However, because context is captured in Equation 1, semantic vectors from Equation 3 that share similar contexts will be located closer together in the vector space. For example, we may find that the context of resistive screen is similar to the contexts of engineered attributes from User Interface, with overlapping words in the context window semantic vector for resistive screen will then be located closer to the engineered attributes from User Interface than from Hardware Specifications.

Because θ is very large and its size depends on the size of the corpus, a naive estimation procedure using iterative optimization techniques is almost always computationally impractical. As such, an efficient approximation algorithm called “negative sampling” is applied (for details, see Gutmann and Hyvärinen [2012]). The central idea is that if the model is trained correctly, it should be good at distinguishing word-context (w,c) pairs observed in our review data from randomly generated pairs $\left(w,{c^{\prime }}_{i}\right)$ ; in other words, ${c^{\prime }}_{i}$ is a “negative sample,” a context generated randomly. Mikolov, Chen, et al. (2013) explicitly compare the skip-gram-negative-sampling method with estimation based on LDA and show that the latter is slow on large data sets due to the Bayesian estimation involved.

**Figure 3.**
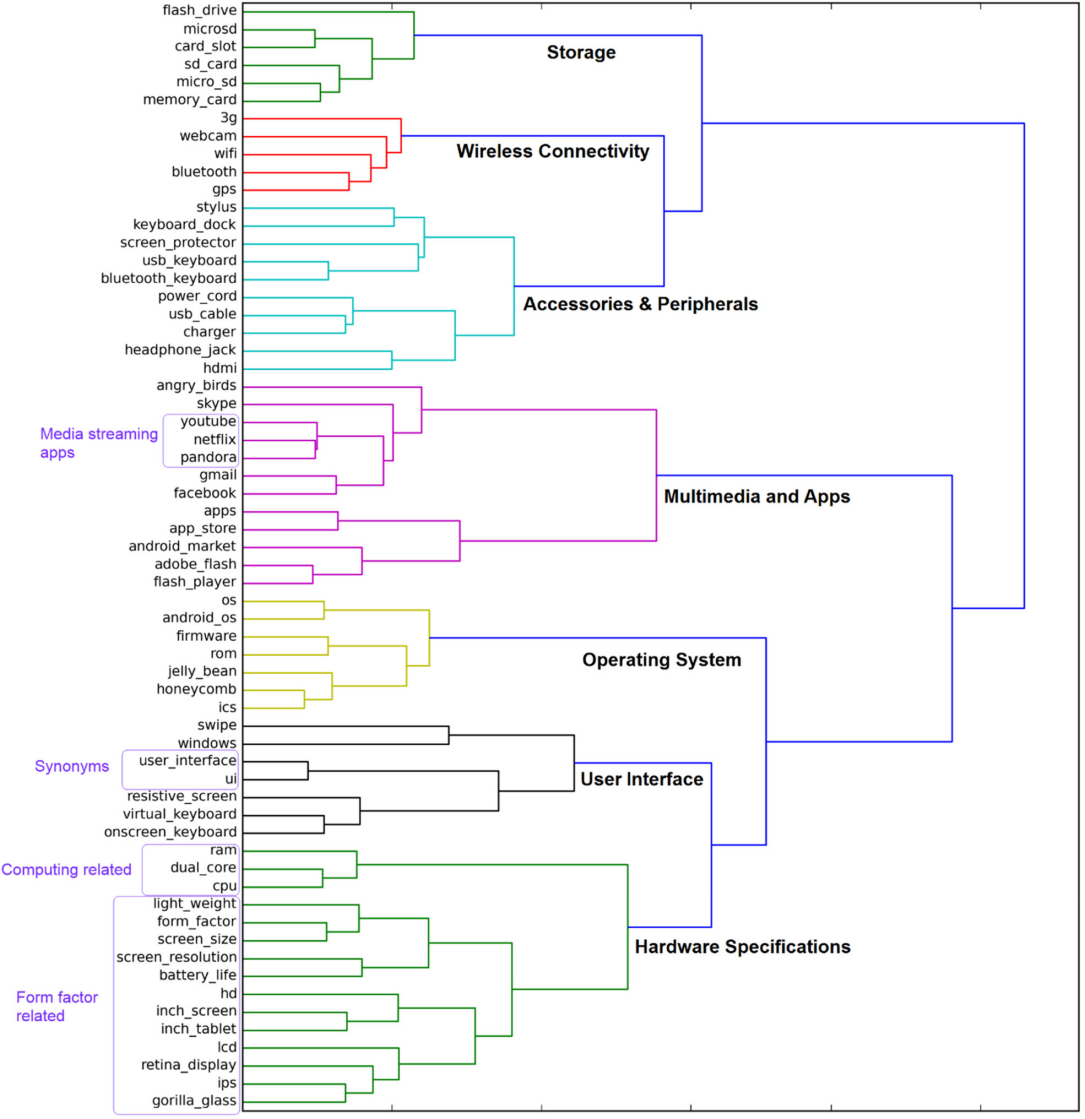
Dendrogram of tablet attribute hierarchy with meta-attributes.

Once the semantic vectors for each engineered attribute have been estimated, we compute the pairwise similarity between all pairs of attributes using the cosine similarity measure defined as $\frac{v_{w1}^{T}v_{w2}}{v_{w1}v_{w2}}$ , where v_w1_ and v_w2_ are the semantic vectors corresponding to attributes w_1_ and w_2_. The similarity matrix then serves as the input for a hierarchical clustering procedure used to construct an attribute hierarchy like the one illustrated in Figure 3. Hierarchical clustering has been widely applied in related tasks such as product categorizations (Srivastava, Leone, and Shocker 1981), product attribute hierarchy construction (Lee and Bradlow 2011), and ontology learning from text (Buitelaar, Cimiano, and Magnini 2005). As part of our methodological framework, hierarchical clustering reveals which engineered attributes cluster to form meta-attributes.

As previously noted, each engineered attribute that forms a meta-attribute may differ in valence. Therefore, our framework incorporates hierarchical sentiment analysis, which uses the context uncovered for an engineered attribute in a given review to predict the sentiment associated with the attribute expressed by that reviewer. When reviewers evaluate a given engineered attribute (e.g., of a tablet), each reviewer essentially casts an implicit “vote” (positive, negative, or neutral) on that attribute. Employing a polarity index yields consistent results when aggregating individual votes from reviews to the brand level and supports aggregation to alternative levels depending on managerial need, such as over SKUs in a product series or over multiple brands. For example, we can aggregate valence by consumer types, by review source, by time period, and in many other ways not explicitly illustrated in this research.

### Implementation for the Tablet Category

We implement our attribute embedding model to analyze customer reviews of tablets. The tablet category has evolved to fulfill increasingly complex consumer needs, illustrating Yoffie’s (1996) notion of digital convergence. Tablets comprise so many engineered attributes that manufacturers face a daunting challenge when determining the right mix. Indeed, several traditional PC manufacturers entered this market only to suffer disappointing results. For example, when HP recognized that features of its flagship tablet, the TouchPad, were poorly received by consumers, the company halted production less than two months after the TouchPad's launch and sold the remaining inventory at deep discounts (Sloane 2011).

To develop managerial insights that can help avoid such debacles, we collected 88,901 online reviews for tablets in March 2014 from Amazon, using the program provided by Wang, Mai, and Chiang (2014). We excluded Kindle Fire reviews because Amazon is the dominant channel of distribution for these tablets and hosts a disproportionate number of product reviews, which might distort the product attribute identification process and bias sentiment scores. The data set includes 306 brands and 1,503 distinct SKUs. Reviews contained an average of 9.14 sentences with 15.4 words on average per sentence. In total, we analyzed 736,224 review sentences and 11,337,851 words. In the engineered attribute extraction step, NLP techniques enable us to infer the set of the most salient product attributes from review sentences. Our algorithm improves on Hu and Liu’s (2004) paradigm for mining consumer opinions by applying a set of filters on the most common nouns and noun phrases. Stop words such as “I,” “the,” “was,” and “a” are filtered out of each noun phrase. The most frequently mentioned noun phrases become candidates for product attributes. These candidates typically exhibit three key problems that we resolve in subsequent steps: (1) redundant nouns that are parts of other noun phrases (e.g., “life” is a redundant noun in the noun phrase “battery life”); (2) noun phrases that are not specific to the product category of interest (i.e., tablets), such as “something,” “people,” “fact,” “others,” or “today”; and (3) noun phrases that are brand names or general product categories, such as “iPad,” “Samsung,” “tablet,” and “tablet computer.” For the technical details for each step, see Web Appendix B.

#### Attribute hierarchy

After the data cleaning and attribute extraction described previously, we implemented the attribute embedding algorithm using a context window size of k = 5 to estimate semantic vectors with dimension d = 100.^1^ The algorithm learned embedded representations for 62 engineered attributes and their corresponding semantic vectors, shown at the base of the dendrogram in Figure 3. A similarity matrix was constructed based on the cosine distance between the semantic vectors. A hierarchy was then generated by agglomerative clustering using this matrix.

We can choose a specific number of meta-attributes by cutting the dendrogram at the appropriate level. We note that distinct managerial objectives pursued by different corporate teams may necessitate different qualitative criteria for choosing the number of meta-attributes. For our study, the selection of the number of meta-attributes is driven primarily by the data for demonstrative purposes. Specifically, we chose seven meta-attributes for three reasons. First, the quantitative evidence indicates that the seven-cluster solution is most “natural” and fits the data well (for details, see Web Appendix C). Second, we validated the seven meta-attributes by comparing our results with those from expert guides and primary research with independent samples of consumers. Third, tests of robustness against alternative specifications with four and ten meta-attributes support the seven-meta-attributes solution.

Although the attribute embedding model does not explicitly generate labels for the meta-attributes, the analyst can conduct a post hoc analysis to review the quality of any labels that are proposed using human judgment. Table 2 summarizes the engineered attributes associated with each meta-attribute.

**Table 2.** Engineered Attributes Associated with Seven Meta-Attributes.

**Meta-Attributes**	**Engineered Attributes**
**Storage**	flash_drive, microsd, card_slot, sd_card, micro_sd, memory_card
**Wireless Connectivity**	3g, webcam, wifi, bluetooth, gps
**Tablet Accessories & Peripherals**	stylus, keyboard_dock, screen_protector, usb_keyboard, power_cord, usb_cable, charger, headphone_jack, admi
**Multimedia & Apps**	angry_bird, skype, youtube, Netflix, pandora, gmail, facebook, apps, app_store, android_market, adobe_flash, flash_player
**Operating System**	os, android_os, firmware, rom, jelly_bean, honeycomb, ics
**User Interface**	swipe, windows, user_interface, ui, resistive_screen, virtual_keyboard, onscreen_keyboard
**Hardware Specifications**	dual_core, cpu, light_weight, form_factor, screen_size, screen_resolution, battery_life, hd, inch_screen, inch_tablet, ldc, retina_display, ips, gorilla_glass

#### Sentiment analysis

To estimate the meta-attribute sentiment scores for each brand, we first require sentiment scores at the sentence level. Using the reviews for 14 tablet brands that garnered the most reviews, we predicted the sentiment (positive, negative, or neutral) of each sentence in our review data. Two human coders manually labeled 2,000 randomly sampled sentences as positive, negative, or neutral. The percentage agreement between the two human coders was 84%, with interrater reliability of κ = .81 (Cohen 1968)—a satisfactory result given the inherent ambiguity of language in free-form reviews. Using the labeled review sentences as a training sample, we predicted the sentiment of the remaining unlabeled sentences in our data using the support vector machine (SVM).^2^ Two classifiers were trained: one to detect positive sentiment and the other to detect negative sentiment. We used bagging (Breiman 1996) to enhance predictive accuracy by training each individual classifier using 15 rounds of bootstrapped samples. The majority vote of the 15 rounds for that classifier can be used to make a prediction.

The sentence-level sentiment score is assigned to an engineered attribute if it has a cosine similarity with the sentence of above .6. Similarity between attribute and sentence is defined as the maximum similarity between attribute and every word in that sentence. A high similarity means the sentence describes something similar to the engineered attribute since semantically similar words are located close together. Therefore, an engineered attribute does not need to be literally mentioned in the sentence. For each meta-attribute, if any of its attributes has a cosine similarity with the sentence of above .6, the sentence sentiment score is assigned to that meta-attribute.^3^ Sentiment scores are then aggregated up to the brand level for all seven meta-attributes using the polarity index. For this process, we define polarity using (P_ij_ − N_ij_)/T_ij_, where P_ij_ is the number of review sentences with positive sentiment, N_ij_ is the number with negative sentiment, and T_ij_ is the total number of sentences that contain meta-attribute j in a brand i. We investigated other polarity measures, such as P_ij_/(N_ij_ + P_ij_) and P_ij_/T_ij_. The results are robust to the selected measures.

### Validations and Robustness Checks

In this section, we conduct a series of validations and robustness checks for our attribute hierarchy using primary and secondary data. Figure 2 references an overview of the tests.

#### Expert guides

To evaluate our attribute hierarchy, we compared our seven meta-attributes with the attributes of tablets suggested in four expert buying guides: Epinions, *Consumer Reports*, Amazon, and eBay (referenced in Figure 2 as RV1). Similar to Lee and Bradlow (2011), we verified whether our meta-attributes not found in expert guides, and conversely, whether attributes found in expert guides, were recovered using our methodological framework. Panel A in Table 3 shows that our framework identifies all the meta-attributes that the expert guides use, but the expert guides miss attributes that we uncover. Panel B quantifies the results using two indices, precision (P) and recall (R) (Salton and McGill 1983).^4^ Our framework demonstrates perfect recall relative to all four expert guides and exhibits better precision than every expert guide with all precision indices exceeding .5. This finding means that all meta-attributes (i.e., abstract product benefits) listed in the expert guides are recovered by our proposed method along with a list of engineered attributes associated with each meta-attribute. In summary, high but not perfect precision indicates that our proposed method uncovers some meta-attributes that are missed in the expert guides.

**Table 3.** Comparison of Meta-Attributes and Attributes from Expert Guides.

**Our Results**	**Epinions**	***Consumer Reports***	**Amazon**	**eBay**
**A: Discovered Meta-Attributes Versus Attributes Extracted from Expert Guides**				
Storage	Gap	Gap	Storage	Storage
Wireless Connectivity	Network type, wireless capabilities	Wireless connectivity	Connectivity	Gap
Tablet Accessories & Peripherals	Input method	Printing capability USB ports	Gap	Keyboard accessories
Multimedia & Apps	Audio output, audio input	Gap	Gap	Gap
Operating System	Platform, OS	OS	OS	OS
User Interface	Supported file types, display tech	Display	Gap	Gap
Hardware Specifications	Gap	Screen size and shape	Screen size	Screen size, processor type
**B: Precision and Recall of Our Method Versus Expert Guides^a^**				
Precision (P)	.71	.71	.57	.57
Recall (R)	1.00	1.00	1.00	1.00

^a^ Precision is the fraction of the seven meta-attributes extracted by our method that are also identified in the expert guide. Recall is the fraction of the meta-attributes identified in the expert guide that are extracted by our method as well.

#### Surveys

To evaluate whether our attribute hierarchy approximates customers’ underlying review-writing behaviors, we conducted several online surveys (RV2 in Figure 2). First, we conducted an exploratory survey on Amazon Mechanical Turk (MTurk), in which we recruited 101 participants. These participants had at least 95% approval ratings for their previous tasks, lived in the United States, and owned a tablet computer. This survey consisted of two tasks. In the first task, for each participant, we randomly selected three meta-attributes uncovered from our attribute hierarchy and presented all their associated engineered attributes in random order. We then asked participants to write a three-paragraph review for the tablet computer that they personally owned, with each paragraph containing at least two sentences that together mentioned at least two of the engineered attributes provided. We then calculated the correlations between the reviewed attributes using phi coefficients to plot a correlation network, assuming that engineered attributes reviewed in the same paragraph are more likely to share implicit relationships than attributes that belong to separate paragraphs.

In the correlation network, we find four clusters that closely resemble the four meta-attributes found in our attribute hierarchy: Hardware Specifications, Accessories & Peripherals, Storage, and Multimedia & Apps. We find that the engineered attributes associated with the remaining three meta-attributes did not receive enough mentions from survey participants to create clear and independent clusters (for details, see Web Appendix E, Survey 1).

In the second task of the survey, we presented participants with the names of all seven meta-attributes found in our attribute hierarchy. They were instructed to list any three concrete features or specifications of tablets that came to mind after reading the name of each meta-attribute. After aggregating the responses, we created word clouds for each meta-attribute based on word frequency. We find that the most frequently mentioned attributes in each word cloud resemble those uncovered in our attribute hierarchy, providing face validity for our method. However, we note that the word clouds also show numerous less frequently mentioned attributes provided by the survey participants; this highlights the drawback of using survey-based methods to elicit concrete features, as it is difficult to determine which of the less frequent features are worth considering. Because our attribute embedding model captures the contexts of how attributes are used in reviews, information from less frequently used words is incorporated in clustering and sentiment analysis (for details, see Web Appendix E, Survey 1).

To evaluate the quality of relationships between engineered attributes and meta-attributes in a more confirmatory manner, we conducted two additional surveys. The surveys asked respondents to perform complementary tasks: (1) to group engineered attributes to form meta-attributes; and (2) given several meta-attributes, to assign each engineered attribute to the meta-attribute where it fits best. In the first survey, we examined whether our uncovered meta-attributes reflected how consumers would group the engineered attributes. We recruited n = 201 participants from MTurk (all U.S. based; rated ≥ 95%) and showed each respondent a list of nine engineered attributes (three engineered attributes randomly selected from each of three randomly selected meta-attributes). Next, participants were instructed to categorize the engineered attributes into three unlabeled groups by considering their similarities in terms of benefits and tablet functionality. We then compared the participants’ coded categories with the meta-attributes from our attribute hierarchy to calculate matching accuracy. We find an overall accuracy of 78.8%, indicating an adequate level of agreement between consumer judgment and the meta-attributes from our attribute hierarchy (for details, see Web Appendix E, Survey 2).

The third survey examined the extent to which each of the engineered attributes is viewed as belonging to a meta-attribute. We recruited n = 179 individuals via MTurk (all U.S. based; rated ≥ 95%) and showed each participant three randomly selected meta-attributes along with ten randomly selected engineered attributes. Participants were asked to evaluate how well an engineered attribute corresponded to the meta-attribute on a six-point scale (0 = “no correspondence at all,” and 5 = “full correspondence”). Results show that consumers view the correspondence in a manner consistent with the attribute hierarchy from our attribute embedding model (for details, see Web Appendix E, Survey 3).

#### Comparison to LDA

We also compared our method with that of Tirunillai and Tellis (2014), which uses an LDA approach to extract topics and topic valence to create brand maps from customer reviews (RV3 in Figure 2). LDA is an unsupervised algorithm, and its bag-of-words assumption presupposes that words observed in customer reviews are independent of each other. Next, we examine whether this characteristic of LDA provides insights that are not revealed by our attribute embedding model.

Table 4 shows the latent topics extracted from our review data using LDA. We varied the number of topics from three to ten. Using six topics minimized perplexity, the geometric mean of the inverse likelihood of observing each word in a held-out data set (Hoffman, Blei, and Bach 2010). As we expected, LDA uncovers informative topics related to tablets. However, without an attribute hierarchy, LDA cannot link engineered attributes to multilevel product benefits. For example, Topic 1 in Table 4 is associated with the phrases “touch,” “touch screen,” “power,” “open,” and “item.” Touch screen and power are engineered attributes that serve quite different functions. Their relationship as viewed by consumers would puzzle analysts—why are these attributes placed in the same topic? Moreover, LDA can be sensitive to whether a specific market is characterized by attributes that are either objectively or subjectively evaluated by consumers. For instance, most consumers agree that objectively evaluated attributes, such as durability and speed, increase utility (i.e., “more durability” and “faster” yield higher quality). Yet, for subjectively evaluated attributes, such as color and style, the notion of “higher quality” is likely to be idiosyncratic, resulting in high variance in sentiment that can exacerbate the problem of assigning valence to topics. In summary, we agree with Tirunillai and Tellis (2014) that LDA is best suited as a strategic tool to identify broad topics of quality in a product category. Our framework, given its drill-down capabilities, can serve as a complement to LDA when analyzing market structures.

**Table 4.** Dimensions Extracted from Our Tablet Review Corpus Using LDA.

**Dimension/Topic**	**Representative Phrases**
1	touch, touch screen, power, open, item
2	kid, wife, learn, card, free
3	movie, full, application, install, add
4	version, access, website, call, awesome
5	cheap, send back, customer service, replace, hour
6	light, OS, hand, amazing, hold, compare, performance

#### News articles and forums

We compared our results with those from Netzer et al.’s (2012) comention approach (RV4 in Figure 2). However, because tablets constitute a relatively new product category, we were unable to find publicly available brand-switching or industry-wide data for a direct comparison. Thus, we resorted to two proxy measures based on (1) the Factiva database, which tracks articles published by top media outlets, and (2) the online forum “What Tablet PC Should I Buy?” hosted by tablet-pcreview.com. We searched these two sources from April 1, 2010, to July 31, 2012, which matches the time window of our analysis.

For the Factiva database, we used the keyword “tablet” and each manufacturer's stock ticker to formulate search queries, following previous research by Gnyawali and Park (2011). We used forum discussion data to calculate a brand co-occurrence measure to replicate the analysis in Netzer et al. (2012). Doing so revealed that brand comentions in a discussion forum provide a proxy for brand switching. The forum contains more than 5,171 threads and 33,856 messages from customers discussing their tablet choices, features, and options. The data set covers brands that consumers consider when making purchase decisions—that is, their consideration set (Hauser and Wernerfelt 1990). It therefore provides a more specific measure of consumer perceptual space than the general forum discussions analyzed by Netzer et al. (2012).

To generate our similarity measure, we used the information theoretic construct lift(A,B)=P(A,B)/[P(A)×P(B)] , where P(A) is the proportion of documents containing brand A returned by the Factiva query or forum search, and P(A,B) is the proportion of documents containing both brands A and B. We obtained an 11 × 11 lift matrix by excluding smaller manufacturers with few mentions. Results using our attribute embedding method correlate well with results from both brand co-occurrence matrices using Factiva query (r = .633; p≤.029 ) and forum search (r = .601; p<.001 ). The correlation between the two external measures is r. 763; p<.001 . Thus, our results converge with those from a published comention method and complement the latter by providing novel insights via drill-down into the nature of competition between pairs of brands.

#### Market share and sales

Because marketing efforts are designed to increase sales and market share, we validated our sentiment analysis results using these bottom-line outcomes (RV5 in Figure 2). We calculated the average market share in the United States in 2012 of each brand, then regressed the averages on the meta-attribute sentiment scores.^5^ The meta-attribute sentiment scores explained 75.2% of the variation (R^2^ = .752). We also validated the results using sales rank data from Amazon (Wang, Mai, and Chiang 2014). Log sales rank is a well-accepted proxy for sales volume and has been used in previous studies (Archak, Ghose, and Ipeirotis 2011; Chevalier and Mayzlin 2006). We calculated the average sales rank of each brand and took the logarithm of the average. The meta-attribute sentiment scores explain 60.4% of the variations in the log sales rank (R^2^ = .604). We compared the results with two other methods; the first calculates sentiment scores using the frequency count of meta-attributes, and the second combines LDA with sentiment analysis. LDA sentiment scores explain 63.5% and 52.2% of the variation in market share and sales, respectively. Basing the meta-attributes on frequency counts is the worst method among these three, explaining only 49.3% and 19.8% variation in the market share and sales, respectively. The results suggest that our method produces meta-attribute sentiment scores that explain considerable variation in market share and sales rank.

#### Alternative tools for sentiment analysis

We benchmarked the robustness of our sentence-level sentiment scores against those from two widely used off-the-shelf sentiment analysis tools: Evaluative Lexicon 2.0 and SentiStrength (RV6 in Figure 2). We examined the correlations between the sentiment scores from these two methods and the results from our positive and negative valence classifiers, yielding correlations of .64 and .55 with Evaluative Lexicon 2.0 and .38 and .37 with SentiStrength. For each sentence, SentiStrength yields a pair of sentiment scores—one for positive valence and one for negative valence—on a scale from 1 to 5. We find that SentiStrength assigns over 50% of our review sentences positive and negative scores of equal magnitudes. The resulting neutral summaries of valence are uninformative and probably explain the relatively low correlations between our sentiment analysis results and SentiStrength.

## Applications of Attribute Hierarchy

We first examined the attribute hierarchy in Figure 3 for qualitative insights. We observe multilevel clusters where engineered attributes within a cluster (and clusters within a higher-level cluster) are expressed in similar contexts by consumers. Lower-level clusters are associated with more specific contexts. For example, the cluster labeled in Figure 3 containing YouTube, Netflix, and Pandora implies that these attributes are often expressed in the contexts of media streaming. Each of our meta-attributes can also be broken down into lower-level clusters (e.g., Hardware Specifications can be broken down into computing-related and factor-related clusters). Lower-level clusters can be useful when managers drill down to analyze a specific meta-attribute. Analysis can also move higher than our level of seven meta-attributes; for instance, Wireless Connectivity and Accessories & Peripherals form a cluster that appears to be related to external connectivity, encompassing the tablet's connections to both wireless networks and physical accessories. However, as clusters become increasingly high level, the ideas that they represent become more abstract and challenging to interpret clearly. Thus, as noted, multiple factors should be considered when selecting the number of meta-attributes.

Our attribute hierarchy can assist various corporate teams. For example, different customer segments may value meta-attributes differently; professionals may value Hardware Specifications more than casual users that value Multimedia & Apps. To target a specific customer segment, the sales team should focus on monitoring and promoting the engineered attributes associated with the target meta-attributes. For new product development, the product design team can examine meta-attributes that are poorly received in previous models and communicate their constituent-engineered attributes to the engineering team as the focus of research and development. As an illustration, we examined the three generations of Apple iPads (iPad 1, iPad 2, and iPad 3). We find that positive sentiments for Wireless Connectivity substantially increased from iPad 1 to iPad 2, driven by the addition of cameras that enabled consumers to use FaceTime launched in June 2010, a fact confirmed by external reports (O’Boyle 2019). However, we observe relatively negative sentiments for “Operating System” (OS) and Hardware Specifications, highlighting areas of improvement for the product design team, and engineered attributes that require refinement to the engineering team. In accordance with these weaknesses, the subsequent iPad 3 exhibits the most positive sentiments for OS and Hardware Specifications, driven by its Retina display (four times the pixels of iPad 2), A5X processor (vs. A5 of iPad 2), and iOS 5 (vs. iOS 4 of iPad 2). For further product series analysis, see Web Appendix F.

### Perceptual maps

**Figure 4.**
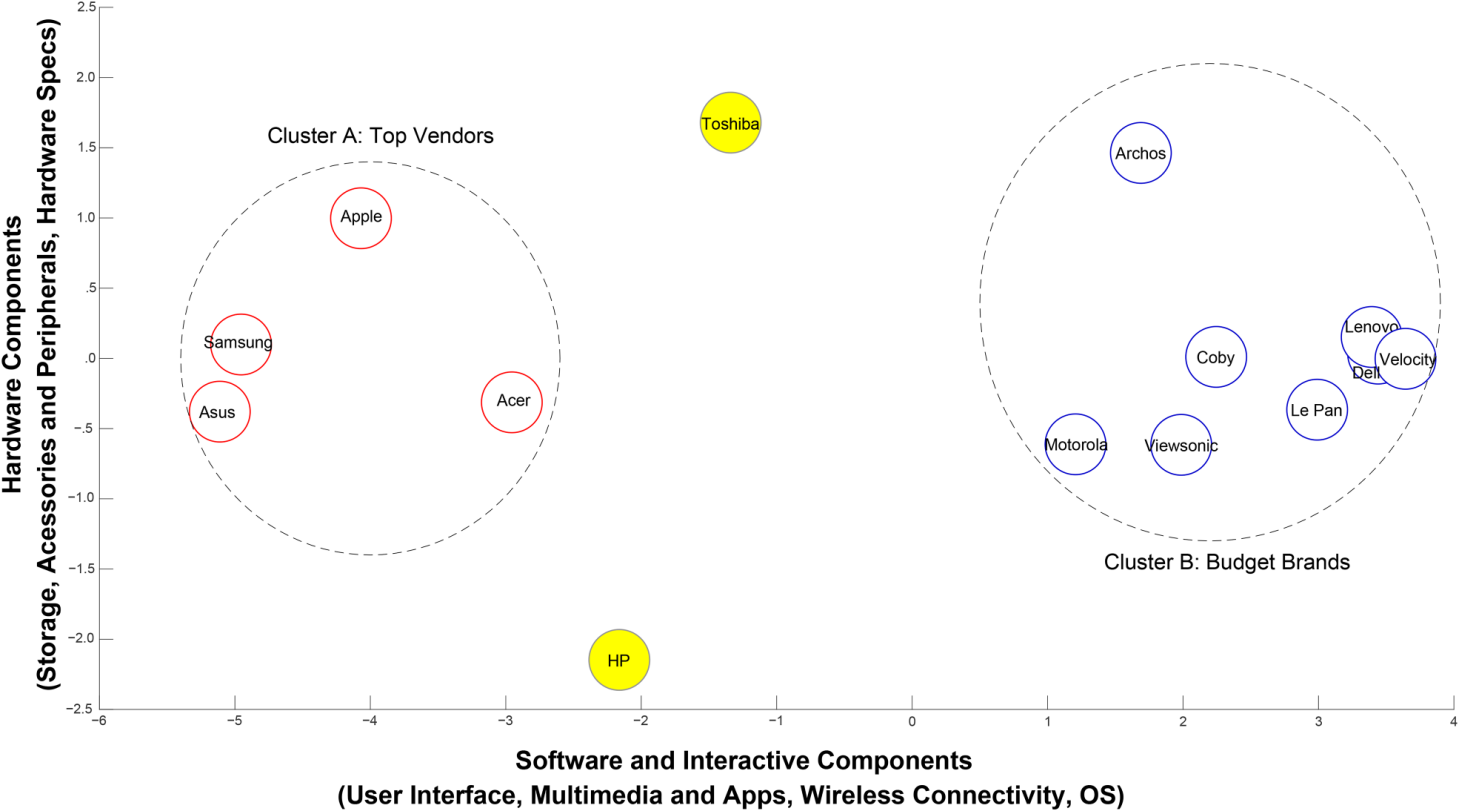
Product positions for 14 brands using consumer sentiment.

To obtain a brand-level perceptual map, we standardized the brand-level meta-attribute sentiment scores to range from 1 to 5, with 5 being the most positive. Pairwise Euclidean distances between brands were then calculated as input to generate a nonmetric multidimensional scaling map at the brand level (Figure 4).

The brand locations in Figure 4 exhibit high face validity for the following reasons. First, most “Budget Brands” cluster together on the right side of the map. These brands are manufactured by companies whose main products are not computers, except for Dell and Lenovo. Accordingly, we find that the average list price of these brands on Amazon is $218 during the study period (Wang, Mai, and Chiang 2014). Second, Cluster A, labeled “Top Vendors,” contains the distinguished leading brands based on worldwide shipments in 2011 and 2012 and exhibits an average list price on Amazon of $592 (IDC 2012). Third, HP is the only one of these manufacturers to use its own OS (WebOS). The market recognizes this distinction and locates HP distant from other brands.

We stepwise regressed brand coordinates on meta-attribute sentiment scores to obtain an approximate interpretation of each axis. The highest loadings for axis 1 correspond to User Interface, Multimedia & Apps, Wireless Connectivity, and OS, which we summarize as “software and interactive components.” Axis 2 corresponds to “Hardware Components,” comprising Storage, Accessories & Peripherals, and Hardware Specifications. Brands show much less differentiation between axis 2 and axis 1, which seems reasonable given that the underlying hardware to create a tablet is quite similar across brands. This gap suggests an opportunity for tablet manufacturers to enhance consumer experience through fundamental innovations at the hardware level. It also highlights that software—or, perhaps more importantly, the integration of software and hardware—is the key to top vendors’ success.

To test the robustness of our map, we compare it with maps generated using four other combinations of techniques. Clarity and precision are important aspects of perceptual maps for managers. Our framework, using hierarchical sentiment scores, uncovers two clear clusters as per Figure 4. This result contrasts with those obtained using alternative methods, which reveal a noisy version of Cluster A and obscure Cluster B altogether (for details, see Web Appendix G).

We used our attribute hierarchy to drill down into the competition between Apple, Samsung, and Dell in one analysis and between Toshiba and HP in another. We selected this set of brands for three reasons. First, Apple and Samsung deserve attention as incumbent leaders in the tablet market. Second, changes in market structure are particularly dramatic in Dell's case, as our analysis reveals. Third, HP and Toshiba held unique brand positions in the tablet market but experienced a lack of success that eventually led to inventory clearance and market exit.

**Figure 5.**
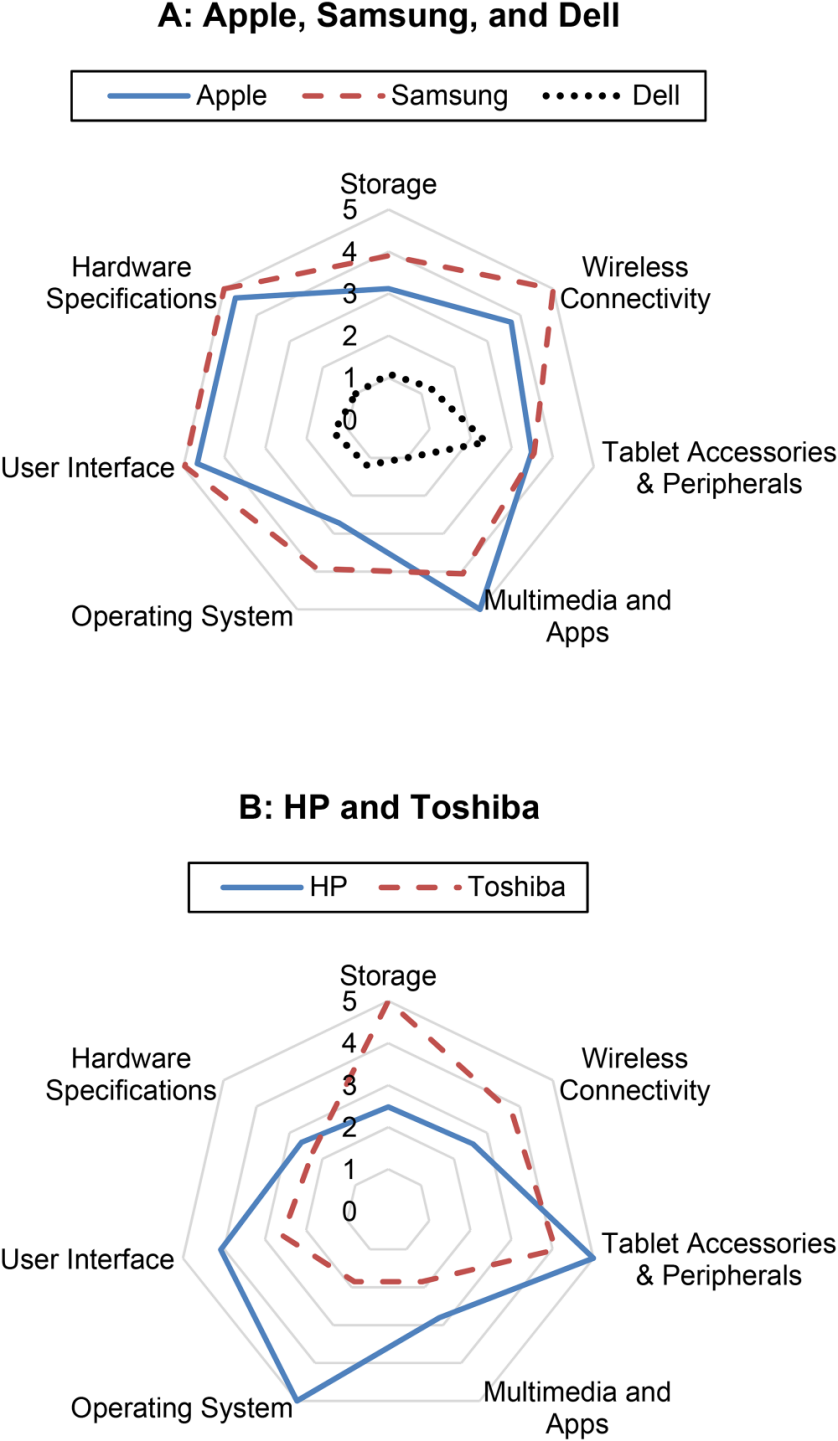
Comparison of meta-attribute sentiment scores on selected brands.

*Notes:* Sentiment scores are standardized to the range of 1 to 5. The plot center corresponds to the zero point.

In Figure 5, Panel A, Apple and Samsung exhibit high scores on all dimensions. Apple has an advantage on Multimedia & Apps (Segan 2012), while Samsung has an advantage on Connectivity. We include Dell to illustrate the stark contrast between these market leaders and a well-known budget brand. Dell's sentiment scores are lackluster on all meta-attributes.

Figure 5, Panel B, features HP and Toshiba, the two brands positioned outside the major clusters in Figure 4. As Figure 5 shows, consumers gave high marks to HP for its OS. Industry experts support this finding, asserting that HP's proprietary WebOS was unique in offering true multitasking and app-switching abilities (Gruman 2011). However, HP's tablet had a “very small number of WebOS apps,” which eventually led to its demise and inventory clearance. Figure 5 also shows Toshiba's competitive advantage in Storage and Accessories & Peripherals. As one *Wall Street Journal* article points out, Toshiba's tablet stood out from its competitors by offering a full-sized SD card slot, a USB port for external storage, and an HDMI port (Mossberg 2011). However, these attributes made the tablet thick and heavy. Furthermore, Toshiba's low score on Multimedia & Apps is echoed in industry reports (Pierce 2012). Congruence between industry experts and our results is encouraging and sends a clear message to tablet manufacturers: identify and rectify meta-attribute weaknesses or face a fate similar to that of Toshiba and HP, both of which discontinued their tablet lines soon after the time window of our analysis.

We then used our attribute hierarchy to drill down to the level of engineered attributes to discover the extent to which each engineered attribute is affecting the sentiment of meta-attributes. Apple dominates in Multimedia & Apps with the highest sentiment scores on all corresponding engineered attributes except Adobe Flash and Flash Player. This finding is intuitive because Apple devices never officially supported Adobe Flash, a fact publicly stated in Steve Jobs’s (2010) open letter, “Thoughts on Flash.” On drilling down into the data, the results for Apple on Accessories & Peripherals are mixed. Consumers liked the charger, power cord, and screen protector, but they expressed negative feelings about HDMI and keyboard dock. Similarly, Samsung has a dominant advantage on Connectivity, achieving the highest sentiment scores on two engineered attributes and the second highest on the remaining three.

In a parallel analysis, we also evaluated brand performance based on negative sentiment. We used the ratio N_ij_/T_ij_ to index negative sentiment, where N_ij_ is the number of sentences with negative sentiment and T_ij_ is the total number of sentences that contain meta-attribute j for brand i. Not surprisingly, Apple dominates. It has the lowest scores (lower is better) on three meta-attributes: Accessories & Peripherals, Operating Systems, and User Interface. On the remaining four meta-attributes, Apple's scores are lower than most brands. As one expert reports, “Apple's complete integration of hardware, software, operating system, and applications is a major piece of what makes the device a standout” (Whitney 2010). The uniformly low negative sentiment indicates that Apple's iPad has no real deal-breakers for consumers. For within-brand analysis, we demonstrate how our attribute hierarchy can analyze successive generations of products (for details, see Web Appendix F).

### Sales forecasting

Previous marketing literature is unequivocal that customer reviews have a positive association with product sales. Although star ratings are commonly used as predictors in sales forecasting models, how review sentiment should be measured to increase predictive performance is less clear. To illustrate how our attribute hierarchy can guide the creation of new predictors, we use the panel of tablet-level weekly sales rank data (Wang, Mai, and Chiang 2014), randomly sampling to use 90% of the data as training sample and the remaining 10% as holdout sample. We focus on R-squared and root mean squared error (RMSE) in the holdout sample as our predictive performance metrics.

We construct our benchmark forecasting model using weekly mean review ratings, the number of weeks the tablet has been on the market, and dummies for tablet brands as predictors (column 1 of Table 5). Calculating the mean is often the most straightforward approach to create predictors using review ratings. In line with this approach, we calculate the weekly mean of review sentiment as an additional predictor to the benchmark model (column 2 of Table 5). We find that this new predictor slightly lifts the R-squared by .006, but also raises RMSE by 6.7 sales ranks. Taken together, the weekly mean of review sentiment provides little improvement in prediction, suggesting that an unguided and crude approach to incorporate sentiment analysis into sales forecasting models may not yield benefits.

**Table 5.** Results of Weekly Sales Rank Prediction.

	**Benchmark**	**Mean Review Sentiment**	**Sentiments of Elicited Attributes**	**Sentiments from Attribute Hierarchy**
	**(1)**	**(2)**	**(3)**	**(4)**
Product-level predictors	Mean review rating, Number of weeks on the market, Brand dummies	Mean review sentiment, Mean review rating, Number of weeks on the market, Brand dummies	Mean sentiment of sentences that contain elicited attributes, each for Storage, Wireless Connectivity, Accessories and Peripherals, Multimedia and Apps, Operating System, User Interface, Hardware Specifications, Mean review rating, Number of weeks on the market, Brand dummies	Mean sentiment of sentences that are associated with the meta-attribute, each for Storage, Wireless Connectivity, Accessories and Peripherals, Multimedia and Apps, Operating System, User Interface, Hardware Specifications, Mean review rating, Number of weeks on the market, Brand dummies
**Holdout Sample Measures**				
R-squared	.219	.225	.076	.261
RMSE	2,701.1	2,707.8	2,742.5	2,583.7

To demonstrate a more guided approach to creating predictors using our attribute hierarchy, we calculate the weekly mean sentiment of sentences classified to each of the seven meta-attributes. We include these seven new predictors in the benchmark model (column 4 of Table 5) and find that the new predictors lift the R-squared by .042 and decrease RMSE by 117.4 sales ranks.

In comparison, we use the responses given by the 101 participants of the exploratory survey we conducted (see Web Appendix E, second task of Survey 1) to show that predictors based on frequency rather than review context could adversely affect the prediction. Instead of using the engineered attributes uncovered by our attribute hierarchy, we use the six most frequently mentioned attributes by the survey participants for each of seven meta-attributes and identify sentences in our review data that contain any of the mentioned attributes. We then calculate the weekly mean sentiment of sentences identified previously for each meta-attribute and include the seven new predictors to the benchmark model (column 3 of Table 5). We find that the R-squared decreases by .143 and RMSE increases by 41.4 sales ranks. This finding illustrates the importance of taking into account the context of reviews. Specifically, failure to incorporate contextual information and naively aggregating sentiment based on frequency alone can increase noise within the individual predictors and hinder model predictions.

## Conclusion

In science and industry, insights are often generated by new measurement tools that support unique viewpoints, be they microscopic or macroscopic. Our approach percolates sentiments upward from the microscopic level (i.e., a single person writing a single review about a single device, sometimes focusing on a single attribute) to the market level. Our method, like those used by other pioneering marketing applications of UGC, creates viewpoints that stimulate thought and are diagnostic in ways that conventional viewpoints cannot be. Our approach is grounded in relevant theories from psychology, linguistics, and consumer behavior; it starts from concrete attributes and creates a hierarchical many-to-one mapping that ascends from engineered attributes to abstract benefits. This mapping “leads to much greater flexibility by potentially enabling physical realizations that satisfy specified constraints or are actually implementable” (Carroll and Green 1997, p. 197). Rather than simply indexing the extent to which pairs of brands are similar, our approach diagnostically unravels the value that customers attach to attributes by using attribute-based sentiment scores (Elrod et al. 2002).

Moreover, our approach supports algorithms that systematically identify impactful outcomes, then seeks to explain those outcomes by using more granular data (i.e., drilling down). There are various ways in which we can drill down into data with our framework, including from meta-attribute to engineered attribute, from brand to product series/model, and from all consumers to designated consumer types. For example, Figure 5, Panel A, shows a clear advantage for Samsung over Apple on the meta-attribute of Wireless Connectivity. But if a marketing analyst were to simply present this gap to corporate strategists, the classic schism between marketing and manufacturing would be exacerbated. Product engineers want to know more than “this gap exists”—they want clear directions on how to fix it at an operational level. Our approach connects this gap directly to five key engineered attributes that comprise Wireless Connectivity (i.e., {3g, webcam, wifi, bluetooth, and gps}. Consequently, Apple's engineers would know precisely which elements of their devices explain the gap and could devote their attention to these specific components.

Tracking attribute-specific aspects of the competitive landscape as they unfold over time is crucial for innovation-based products such as tablets (Kim and Kim 2015). With sufficient resources, structural insights can be generated in real time and support automated drill-down to explain how consumer sentiment for a brand is changing. Thus, our approach provides an important complement to scanner-based market share summaries, which offer only indirect evidence of market trends and little, if any, evidence about why they are occurring.

### Limitations

The use of UGC in marketing research is still in its infancy. In many ways, our work is typical of research in an emerging discipline in which the first stage is taxonomic. When analyzed effectively, UGC has great promise, but this rapidly developing area also presents significant challenges.

#### Unknown ground truth

In general, validating the results of unsupervised methods such as word embedding or hierarchical clustering is challenging because the “gold standard” is hard to define (Sabou 2005). At present, analysts can compare solutions from different methods, but there is no universal model selection criterion. For example, our hierarchy and the resulting structural inferences about attributes are not explicitly price-scaled, as suggested by Shugan (2015, p. 150). Nevertheless, hierarchies generated by our method may be implicitly price-scaled because individuals tend to reflect their own income levels in their attitudes about a product when writing reviews.

#### Data availability and quality

As Timoshenko and Hauser (2019) note, UGC data are not available for every product category. In addition, UGC methods work best with very large data sets, but such data sets are difficult to come by, unwieldy, and subject to spam and malicious or fake reviews, which compromise data quality.

#### Need for human intervention

Machine learning techniques for attribute extraction and hierarchy identification currently require some level of human intervention. In our methodological framework, we used human coders to label sentences to train the sentiment analysis algorithms. Additional, nonautomated work was required to properly label meta-attributes, and we set the hyperparameters of our models following previous literature. However, we conducted extensive sensitivity tests to ensure that the results are robust against major variations in these parameters. While it is still possible that tuning hyperparameters in neural net models and in other components of our work may suffer from biases, every NLP-based technique to date faces these same problems. Inevitably, as NLP matures, so will the methods that solve these problems.

### Future Research

Despite the complexities of conducting research with UGC, marketing applications will grow rapidly in the next few years. We outline two promising areas for future research: (1) individual MSA solutions and (2) intertextual dependencies.

#### Proprietary data and individual MSA solutions

Compared with traditional MSA techniques, consumer-generated product reviews can be collected at a much higher frequency and from multiple sources. The present research uses nonproprietary data with minimal amounts of metadata at the individual review level. However, Amazon has purchase history and demographic data for individual reviewers. Similarly, social media giants such as Facebook have millions of product commentaries coupled with rich data about each commentator. Analyses that generate individual market structure maps—in a fashion analogous to the individual-level utility functions generated using choice-based conjoint analysis—represent one challenging but intriguing area for future research to explore. Given today's highly targeted media, a firm could boost customer retention by actively adjusting its brand's advertised position according to how an individual consumer evaluates the brand's functional benefits. MSA at the individual level could also be employed in recommendation systems using the direct link between meta-attributes and engineered attributes (De Bruyn et al. 2008).

#### Intertextual dependencies

Continued advancements in machine learning will enable marketing researchers to uncover increasingly deeper complexities from text. Whereas previous studies in marketing, though seminal, were limited to the use of dictionaries and assumptions about the independence of words, newly developed NLP techniques can model intertextual dependency or relationships between words. In this research, we use the skip-gram model to identify contexts shared between product attributes that appear in customer reviews. Future marketing research on online word of mouth could explore other types of intertextual dependencies beyond product attributes, such as relationships among texts written by consumers segmented according to traditional measures (e.g., demographic, geographic, psychographic). Because text can yield rich information about consumers’ underlying cognitive states (Netzer, Lemaire, and Herzenstein 2019), we believe that intertextual dependency will become an increasingly important source of nuanced managerial insights about different consumer cohorts.

## Supplemental Material

sj-pdf-1-jmx-10.1177_00222429211047822 - Supplemental material for Attribute Embedding: Learning Hierarchical Representations of Product Attributes from Consumer Reviews Supplemental material, sj-pdf-1-jmx-10.1177_00222429211047822 for Attribute Embedding: Learning Hierarchical Representations of Product Attributes from Consumer Reviews by Xin (Shane) Wang, Jiaxiu He, David J. Curry and Jun Hyun (Joseph) Ryoo in Journal of Marketing

## Appendix: Pseudocode

**Table:**

**Prepare Data: Transform Reviews to Words/Phrases in Root Form**		
In	C_R_: set of original text product reviews	
1	For $\mathrm{revie}w_{i}\in C_{R}$ : BREAK to sentences For $\mathrm{sentenc}e_{j}\in \mathrm{revie}w_{i}$ : BREAK to token words For $\mathrm{wor}d_{k}\in \mathrm{sentenc}e_{j}$ : TRANSFORM to root form STORE in C_RW_ ( $C_{R}\to C_{\mathrm{RW}}$ ): reviews in root form	➮ call **sent_tokenize** in **NLTK** ➮ call **word_tokenize** in **NLTK** ➮ call **WordNetLemmatizer** in **NLTK**
2	For $\mathrm{sentenc}e_{j}\in C_{\mathrm{RW}}$ : TAG part-of-speech of each token word	➮ call **TextBlob**
3	For $\mathrm{sentenc}e_{j}\in C_{\mathrm{RW}}$ : IDENTIFY words appear together frequently	➮ call **bigram** in **GenSim**
4	STORE in R ( $C_{\mathrm{RW}}\to R$ ): reviews containing root-form token phrases	
**Extract Engineered Attributes**		
In	R: set of reviews containing root-form token phrases (review corpus) IR: set of reviews for irrelevant products in root-form token phrases	
1	For all reviews in R and IR: Remove stop words	➮ call **stopwords** in **NLTK**
2	RETAIN noun-phrases (NPs) s.t. $\mathrm{Support}(\mathrm{NP})\geq s_{0}$ , ADD to set C For $NP_{j}\in C$ $\mathrm{PureSupport}(NP_{j})\leftarrow \mathrm{Support}(NP_{j})$ For $NP_{k}\in R$ s.t. j≠k If $NP_{j}\subset NP_{k}$ : $PureSupport(NP_{j})=\mathrm{PureSupport}(NP_{j})-\mathrm{Support}(NP_{j})$	➮ calculate pure support
3	For $NP_{j}\in C\cap \mathrm{IR}$ $C_{11}^{j}=\mathrm{Support}(NP_{j})\times |R|$ $C_{12}^{j}=|R|-C_{11}^{j}$ $C_{21}^{j}=\mathrm{Suppor}t_{\mathrm{ir}}(NP_{j})\times |\mathrm{IR}|$ $C_{22}^{j}=|\mathrm{IR}|-C_{21}^{j}$ DEFINE: $r_{1}=\frac{C_{11}^{j}}{C_{11}^{j}+C_{12}^{j}}$ ; $r_{2}=\frac{C_{21}^{j}}{C_{21}^{j}+C_{22}^{j}}$ ; $r=\frac{C_{11}^{j}+C_{21}^{j}}{C_{11}^{j}+C_{12}^{j}+C_{21}^{j}+C_{22}^{j}}$ IF $\mathrm{Suppor}t_{\mathrm{ir}}(NP_{j})>\mathrm{Support}(NP_{j})$ ; $\mathrm{LR}(NP_{j})=0$ ELSE; $\mathrm{LR}(NP_{j})=-2\left[\begin{array}{c} \left(C_{11}^{j}+C_{21}^{j})\log\;r^{j}+(C_{12}^{j}+C_{22}^{j})\log\;(1-r^{j})-C_{11}^{j}\log\;r_{1}^{j}-C_{12}^{j}\log\;(1-r_{1}^{j}\right)\\ -C_{21}^{j}\log\;r_{2}^{j}-C_{22}^{j}\log\;(1-r_{2}^{j}) \end{array}\right]$	➮ s: support threshold (manually set) ➮ s_0_: initialize $(s_{0}\ll s$ , manually set) ➮ p_s_: pure support threshold (pure support is support of a stand-alone phrase, manually set) ➮ calculate likelihood ratio ➮ lr: threshold (manually set)
4	For $NP_{j}\in C$ IF $\mathrm{LR}(NP_{j})\leq \mathrm{lr}$ OR $\mathrm{Support}(NP_{j})\leq s$ OR $\frac{\mathrm{PureSupport}(NP_{j})}{\mathrm{Support}(NP_{j})}$ ≤ps ; REMOVE NP_j_	➮ Filter NPs by Support, PureSupport using the Likelihood Ratio
5	MANUALLY FILTER unrelated NPs from C The resulting C contains 62 Engineered Attributes (i.e. root-form [tokenized] noun-phrases).	
**Construct Attribute Hierarchy**		
In	R: review corpus	
1	HMat: 62×62 matrix d = 100; k = 5	➮ initialize similarity matrix ➮ set dimension, window size
2	TRAIN embedding model, STORE resulting model as M	➮ call **word2vec** in **GenSim**
3	For $\mathrm{Attribut}e_{k}\in C$ such that i≠j CALCULATE $\mathrm{Sim}(\mathrm{At}t_{i},\mathrm{At}t_{j})$ , store in HMat(i,j)	
4	DRAW dendrogram from HMat	➮ call **dendrogram** in **SciPy**
**Predict Valence of Review**		
In	R: review corpus of root-form token phrases T: set of 2,000 training sentences with valence classified by human coders ( T⊂R ) W^P^: a list of positive opinion training words W^N^: a list of negative opinion training words Rating: star rating by review∈R C: set of product attributes of relevant products	
1	For $\mathrm{sentenc}e_{j}\in R$ COUNT positive words in sentence: n_j_ COUNT negative words in sentence: m_j_ CALCULATE $\mathrm{rawsen}t_{j}=n_{j}-m_{j}$ and $\mathrm{mago}p_{j}=\frac{n_{j}-m_{j}}{n_{j}+m_{j}}$	
2	COUNT number of reviews in R: D For $\mathrm{phras}e_{k}\in R$ ADD phrase_k_ to set Z COUNT reviews containing phrase_k_: d_k_ COUNT frequency of phrase_k_: N_k_ CALCULATE $I_{k}=\left(1-\sqrt{d_{k}}/D\right)\left(\sqrt{N_{k}}\right)$ SORT all $\mathrm{phras}e_{k}\in Z$ by index I_k_ RETAIN 200 phrases with largest I_k_ For $\mathrm{sentenc}e_{j}\in R$ IDENTIFY all $\mathrm{phras}e_{k}\in \mathrm{sentenc}e_{j}\cap Z$ CALCULATE $\mathrm{inde}x_{j}=\underset{k}{\sum }\;I_{k}$	➮ Determine domain-specific features of text
3	For b = 1, 2, …, 15 SVM PREDICT $v_{j}^{P,b}(=0\;\;\mathrm{or}\;\;1)$ , SVM PREDICT $v_{j}^{N,b}(=0\;\;\mathrm{or}\;;1)$ PREDICT $v_{j}^{P}=\mathrm{majority}(v_{j}^{P,1},\ldots ,v_{j}^{P,15})$ PREDICT $v_{j}^{N}=\mathrm{majority}(v_{j}^{N,1},\ldots ,v_{j}^{N,15})$	➮ Bootstrap with replacement ➮ Predict valence using rawsent_j_, magop_j_, rating_j_, and index_j_; call **SVM** in **SKLEARN** ➮ Predict valence by voting
	IF $v_{j}^{P}=1$ and $v_{j}^{N}=1$ $\mathrm{CP}=\underset{b}{\sum }\;I(v_{j}^{P,b}=1)$ $\mathrm{CN}=\underset{b}{\sum }\;I(v_{j}^{N,b}=1)$ IF CP < CN: $v_{j}^{P}=0$ ELSE IF CP > CN: $v_{j}^{N}=0$ ELSE: $v_{j}^{P}=0$ ; $v_{j}^{N}=0$	➮ If conflict exists ➮ count of rounds predicted positive ➮ count of rounds predicted negative
	STORE $v_{j}^{P}$ and $v_{j}^{N}$ in V^P^ and V^N^	➮ V^P^, V^N^, valence prediction vectors
4	For $\mathrm{sentenc}e_{j}\in R$ For $EA_{k}\in C$ IF $\mathrm{sim}(\mathrm{sentenc}e_{j},\;EA_{i})\geq .6$ ${\mathrm{sentiment}}_{j}^{P,EA_{k}}=v_{j}^{P}$ ${\mathrm{sentiment}}_{j}^{N,EA_{k}}=v_{j}^{N}$	➮ Calculate sentiments of engineered attribute (EA) in each sentence
5	For $\mathrm{sentenc}e_{j}\in R$ For MA_m_ IF $\mathrm{sim}(\mathrm{sentenc}e_{j},\;MA_{m})\geq .6$ ${\mathrm{sentiment}}_{j}^{P,MA_{m}}=v_{j}^{P}$ ${\mathrm{sentiment}}_{j}^{N,MA_{m}}=v_{j}^{N}$	➮ Calculate sentiments of meta-attribute (MA) in each sentence ➮ $\begin{array}{rl} & \mathrm{sim}(\mathrm{sentenc}e_{j},\;MA_{m})=\\ & \underset{k,EA_{k}\in MA_{m}\;}{\max}\;[\mathrm{sim}(\mathrm{sentenc}e_{j},\;EA_{k})] \end{array}$
6	For brand_i_ For MA_m_ COUNT sentences s.t. $\mathrm{sim}(\mathrm{sentenc}e_{j},\;MA_{m})\geq .6$ in all reviews of brand i product: $T_{i}^{MA_{m}}$	➮ Calculate brand-wise sentiment score of meta-attributes
	For $\mathrm{sentenc}e_{j}\in R\cap \mathrm{bran}d_{i}$ ${\mathrm{sentiment}}_{i}^{P,MA_{m}}=\underset{j}{\sum }\;{\mathrm{sentiment}}_{j}^{P,MA_{m}}$ ${\mathrm{sentiment}}_{i}^{N,MA_{m}}=\underset{j}{\sum }\;{\mathrm{sentiment}}_{j}^{N,MA_{m}}$ ${\mathrm{sentiment}}_{i}^{MA_{m}}$ = $\;\left({\mathrm{sentiment}}_{i}^{P,MA_{m}}-{\mathrm{sentiment}}_{i}^{N,MA_{m}}\right)/T_{i}^{MA_{m}}$	➮ Counts of sentences with positive/negative valence ➮ Overall sentiment score
7	For brand_i_ FOR each $EA_{k}\in C\;$ COUNT sentences s.t. $\mathrm{sim}(\mathrm{sentenc}e_{j},\;EA_{k})\geq .6$ in all reviews of brand i product: $T_{i}^{EA_{k}}$	➮ Calculate brand-wise sentiment score of engineered attributes
	For $\mathrm{sentenc}e_{j}\in R\cap \mathrm{bran}d_{i}$ ${\mathrm{sentiment}}_{i}^{P,EA_{k}}\;=\underset{j}{\sum }\;{\mathrm{sentiment}}_{j}^{P,EA_{k}}$ ${\mathrm{sentiment}}_{i}^{N,EA_{k}}=\underset{j}{\sum }\;{\mathrm{sentiment}}_{j}^{N,EA_{k}}$ ${\mathrm{sentiment}}_{i}^{EA_{k}}$ = $\;({\mathrm{sentiment}}_{i}^{P,EA_{k}}-{\mathrm{sentiment}}_{i}^{N,EA_{k}})/T_{i}^{EA_{k}}$	➮ Counts of sentences with positive/negative valence ➮ Overall sentiment score

*Notes:* NLTK, TextBlob, GenSim, SciPy, and SKLEARN are Python packages used in this study.

## Notes

1 These are default values used in the Python packages. We vary the values of window size and dimension to check the sensitivity of choices. We compare the correlations of pairwise attribute similarities across different choices of parameter values. All correlations are above .95, suggesting that the results are robust.

2 We also used maximum entropy, classification trees, naive Bayes, and random forests. SVM outperformed the others (for details, see Web Appendix D). In addition, SVM significantly outperformed the lexicon approach, which uses a precompiled dictionary of positive and negative words, an approach often used in previous marketing research (Tirunillai and Tellis 2014).

3 Some sentences do have high similarity with multiple engineered attributes, but in over 90% of the cases, these attributes belong to different meta-attributes.

4 Precision is the fraction of the seven meta-attributes extracted by our method that are also identified in the expert guide. Recall is the fraction of the meta-attributes identified in the expert guide that are extracted by our method as well.

5 We use market share data from https://gs.statcounter.com/vendor-market-share/tablet/ (accessed June 24, 2020).

## References

[bibr1-00222429211047822] Archak Nikolay Ghose Anindya Ipeirotis Panagiotis G. (2011), “Deriving The Pricing Power of Product Features by Mining Consumer Reviews,” Management Science, 57 (8), 1485–1509.

[bibr2-00222429211047822] Berger Jonah Humphreys Ashlee Ludwig Stephan Moe Wendy W. Netzer Oded Schweidel David A. (2020). “Uniting the Tribes: Using Text for Marketing Insight,” Journal of Marketing, 84 (1), 1–25.

[bibr3-00222429211047822] Bettman James Luce Mary Frances Payne John (1998), “Constructive Consumer Choice Processes,” Journal of Consumer Research, 25 (3), 187–217.

[bibr4-00222429211047822] Breiman Leo (1996), “Bagging Predictors,” Machine Learning, 24 (2), 123–40.

[bibr5-00222429211047822] Buitelaar Paul Cimiano Philipp Magnini Bernardo (2005), “Ontology Learning from Text: An Overview,” in Ontology Learning from Text: Methods, Evaluation and Applications, Vol. 123, Paul Buitelaar, Philipp Cimiano, and Bernardo Magnini, eds. Amsterdam: IOS Press, 3–12.

[bibr6-00222429211047822] Carroll J. Douglas Green Paul E. (1997), “Psychometric Methods in Marketing Research: Part II, Multidimensional Scaling,” Journal of Marketing Research, 34 (2), 193–204.

[bibr7-00222429211047822] Chen Dawn Lu Hongjing Holyoak Keith (2015), “Learning and Generalizing Cross-Category Relations Using Hierarchical Distributed Representations,” in Proceedings of the 37th Annual Conference of the Cognitive Science Society, D.C. Noelle, R. Dale, A.S. Warlaumont, J. Yoshimi, T. Matlock, C.D. Jennings, et al., eds. Austin: Cognitive Science Society, 339–44.

[bibr8-00222429211047822] Chevalier Judith A. Mayzlin Dina (2006), “The Effect of Word of Mouth on Sales: Online Book Reviews,” Journal of Marketing Research, 43 (3), 345–54.

[bibr9-00222429211047822] Cohen Jacob (1968), “Weighted Kappa: Nominal Scale Agreement Provision for Scaled Disagreement or Partial Credit,” Psychological Bulletin, 70 (4), 213–20. 19673146 10.1037/h0026256

[bibr10-00222429211047822] De Bruyn Arnaud Liechty John C. Huizingh Eelko K.R.E. Lilien Gary L. (2008), “Offering Online Recommendations with Minimum Customer Input Through Conjoint-Based Decision Aids,” Marketing Science, 27 (3), 443–60.

[bibr11-00222429211047822] Elrod Terry Russell Gary J. Shocker Allan D. Andrews Rick L. Bacon Lynd Bayus Barry L. , et al. (2002), “Inferring Market Structure from Customer Response to Competing and Complementary Products,” Marketing Letters, 13 (3), 221–32.

[bibr12-00222429211047822] Gnyawali Devi R. Park Byung-Jin (2011), “Co-opetition Between Giants: Collaboration Between Competitors for Technological Innovation,” Research Policy, 40 (5), 650–63.

[bibr13-00222429211047822] Griffin Abbie Hauser John R. (1993), “The Voice of the Customer,” Marketing Science, 12 (1), 1–27.

[bibr14-00222429211047822] Gruman Galen (2011), “Tablet Deathmatch: HP TouchPad vs. Apple iPad 2,” InfoWorld (June 29), https://www.infoworld.com/article/2622318/tablet-deathmatch--hp-touchpad-vs--apple-ipad-2.html.

[bibr15-00222429211047822] Gustafsson Anders Herrmann Andreas Huber Frank (2007), Conjoint Measurement: Methods and Applications. Berlin: Springer.

[bibr16-00222429211047822] Gutmann Michael U. Hyvärinen Aapo (2012), “Noise-Contrastive Estimation of Unnormalized Statistical Models, with Applications to Natural Image Statistics,” Journal of Machine Learning Research, 13, 307–61.

[bibr17-00222429211047822] Hauser John R. Clausing Don (1988), “The House of Quality,” Harvard Business Review, 66 (3), 63–73.

[bibr18-00222429211047822] Hauser John R. Wernerfelt Birger (1990), “An Evaluation Cost Model of Consideration Sets,” Journal of Consumer Research, 16 (4), 393–408.

[bibr19-00222429211047822] Hoffman Matthew Blei David M. Bach Francis (2010), “Online Learning for Latent Dirichlet Allocation,” in Advances in Neural Information Processing Systems 23, J. Lafferty, C. Williams, J. Shawe-Taylor, R. Zemel, and A. Culotta, eds. NeurIPS: 856–64.

[bibr20-00222429211047822] Howard John A. Sheth Jagdish N. (1969), The Theory of Buyer Behavior. New York: John Wiley & Sons.

[bibr21-00222429211047822] Hu Minqing Liu Bing (2004), “Mining and Summarizing Customer Reviews,” in Proceedings of the Tenth ACM SIGKDD International Conference on Knowledge Discovery and Data Mining. New York: Association for Computing Machinery, 168–77.

[bibr22-00222429211047822] IDC (2012), “Top 5 Vendors, Worldwide Media Tablet Shipments, Second Quarter 2012,” (accessed June 3, 2020), https://sourcedigit.com/wp-content/uploads/2012/08/IDC-Worldwide-Mobile-Phone-Tracker-July-26-2012.jpg.

[bibr23-00222429211047822] Jobs Steve (2010), “Thoughts on Flash,” Open Letter (April), https://newslang.ch/wordpress/wp-content/uploads/2020/06/Thoughts-on-Flash.pdf.

[bibr24-00222429211047822] Johnson Michael D . (1988), “Comparability and Hierarchical Processing in Multialternative Choice,” Journal of Consumer Research, 15 (3), 303–14.

[bibr25-00222429211047822] Johnson Michael D . (1989), “The Differential Processing of Product Category and Noncomparable Choice Alternatives,” Journal of Consumer Research, 16 (3), 300–09.

[bibr26-00222429211047822] Johnson Michael D. Lehmann Donald R. Fornell Claes Horne Daniel R. (1992), “Attribute Abstraction, Feature-Dimensionality, and the Scaling of Product Similarities,” International Journal of Research in Marketing, 9 (2), 131–47.

[bibr27-00222429211047822] Kibbe Melissa M. Feigenson Lisa (2014), “Developmental Origins of Recoding and Decoding in Memory,” Cognitive Psychology, 75 (December), 55–79. 25195153 10.1016/j.cogpsych.2014.08.001

[bibr28-00222429211047822] Kim Dong Soo Bailey Roger A. Hardt Nino Allenby Greg M. (2017), “Benefit-Based Conjoint Analysis,” Marketing Science, 36 (1), 54–69.

[bibr29-00222429211047822] Kim Wonjoon Kim Minki (2015), “Reference Quality-Based Competitive Market Structure for Innovation Driven Markets,” International Journal of Research in Marketing, 32 (3), 284–96.

[bibr30-00222429211047822] Lancaster Kelvin J . (1966), “A New Approach to Consumer Theory,” Journal of Political Economy, 74 (2), 132–57.

[bibr31-00222429211047822] Lee Thomas Y. Bradlow Eric T. (2011), “Automated Marketing Research Using Online Customer Reviews,” Journal of Marketing Research, 48 (5), 881–94.

[bibr32-00222429211047822] Liu Xiao Lee Dokyun Srinivasan Kannan (2019), “Large-Scale Cross-Category Analysis of Consumer Review Content on Sales Conversion Leveraging Deep Learning,” Journal of Marketing Research, 56 (6), 918–43.

[bibr33-00222429211047822] Luce R. Duncan Tukey John W (1964), “Simultaneous Conjoint Measurement: A New Type of Fundamental Measurement,” Journal of Mathematical Psychology, 1 (1), 1–27.

[bibr34-00222429211047822] Melumad Shiri Inman J. Jeffrey Pham Michel Tuan (2019), “Selectively Emotional: How Smartphone Use Changes User-Generated Content,” Journal of Marketing Research, 56 (2), 259–75.

[bibr35-00222429211047822] Mikolov Tomas Chen Kai Corrado Greg Dean Jeffrey (2013), “Efficient Estimation of Word Representations in Vector Space,” in Proceedings of Workshop at ICLR, https://www.semanticscholar.org/paper/Efficient-Estimation-of-Word-Representations-in-Mikolov-Chen/330da625c15427c6e42ccfa3b747fb29e5835bf0.

[bibr36-00222429211047822] Mikolov Tomas Sutskever Ilya Chen Kai Corrado Greg Dean Jeffrey (2013), “Distributed Representations of Words and Phrases and Their Compositionality,” in *Proceedings of the 26th International Conference on* Neural Information Processing Systems. New York: Assocation for Computing Machinery, 3111–19.

[bibr37-00222429211047822] Moon Sangkil Kamakura Wagner A. , (2017), “A Picture Is Worth a Thousand Words: Translating Product Reviews into a Product Positioning Map,” International Journal of Research in Marketing, 34 (1), 265–85.

[bibr38-00222429211047822] Mossberg Walter (2011), “Tablet Strives to Plug into Laptops’ Port Abilities,” *The Wall Street Journal* (July 14), https://www.wsj.com/articles/SB10001424052702303406104576444052144872400.

[bibr39-00222429211047822] Netzer Oded Feldman Ronen Goldenberg Jacob Fresko Moshe (2012), "Mine Your Own Business: Market-Structure Surveillance Through Text Mining,” Marketing Science, 31 (3), 521–43.

[bibr40-00222429211047822] Netzer Oded Lemaire Alain Herzenstein Michal (2019), “When Words Sweat: Identifying Signals for Loan Default in the Text of Loan Applications,” Journal of Marketing Research, 56 (6), 960–80.

[bibr41-00222429211047822] Netzer Oded Toubia Olivier Bradlow Eric T. Dahan Ely Evgeniou Theodoros Feinberg Fred M. , et al. (2008), “Beyond Conjoint Analysis: Advances in Preference Measurement,” Marketing Letters, 19 (3), 337–54.

[bibr42-00222429211047822] O’Boyle Britta (2019), “The Apple iPad Through Time: Over a Decade of iPad Revisited,” Pocket-lint (June 5), https://www.pocket-lint.com/tablets/news/apple/146888-history-of-the-apple-ipad.

[bibr43-00222429211047822] Payne John W. Bettman James R. Johnson Eric J. (1993), The Adaptive Decision Maker. Cambridge, UK: Cambridge University Press.

[bibr44-00222429211047822] Pierce David (2012), “Toshiba Excite Review: 13, 10, and 7.7-Inch Tablets,” *The Verge* (June 18), https://www.theverge.com/2012/6/18/3094316/toshiba-tablet-reviews-2012-excite-13-10-7-inch.

[bibr45-00222429211047822] Sabou Marta (2005), “Learning Web Service Ontologies: An Automatic Extraction Method and Its Evaluation,” Ontology Learning from Text: Methods, Evaluation and Applications, Vol. 123, Paul Buitelaar, Philipp Cimiano, and Bernardo Magnini, eds. Amsterdam: IOS Press, 125–39.

[bibr46-00222429211047822] Salton Gerard Mcgill Michael J. (1983), Introduction to Modern Information Retrieval. New York: McGraw-Hill.

[bibr47-00222429211047822] Segan Sascha (2012), “Windows on ARM vs. iPad: The New Mac/PC War?” *PC Magazine* (February 10), https://uk.pcmag.com/tablets/66482/windows-on-arm-vs-ipad-the-new-macpc-war.

[bibr48-00222429211047822] Shugan Steven (2015), “Market Structure Research,” in The History of Marketing Science, Winer R. Neslin S. , eds. Danvers, MA: World Scientific Publishing Co, 129–64.

[bibr49-00222429211047822] Sloane Garett (2011), “Out of ‘Touch’: HP Exits Tablet Business,” *The New York Post* (August 20), https://nypost.com/2011/08/20/out-of-touch-hp-exits-tablet-business/.

[bibr50-00222429211047822] Srivastava Rajendra K. Leone Robert Shocker Allan (1981), “Market Structure Analysis: Hierarchical Clustering of Products Based on Substitution-in-Use,” Journal of Marketing, 45 (3), 38–48.

[bibr51-00222429211047822] Timoshenko Artem Hauser John R. (2019), “Identifying Customer Needs From User-Generated Content,” Marketing Science, 38 (1), 1–20.

[bibr52-00222429211047822] Tirunillai Seshadri Tellis Gerard J. (2014), “Mining Marketing Meaning from Online Chatter: Strategic Brand Analysis of Big Data Using Latent Dirichlet Allocation,” Journal of Marketing Research, 51 (4), 463–79.

[bibr53-00222429211047822] Wang Xin (Shane) Mai Feng Chiang Roger H.L. (2014), “Database Submission-Market Dynamics and User-Generated Content About Tablet Computers,” Marketing Science, 33 (3), 449–58.

[bibr54-00222429211047822] Whitney Lance (2010), “Report: iPad to Rule Tablet Market Through 2012,” CNET (August 25), https://www.cnet.com/news/report-ipad-to-rule-tablet-market-through-2012/.

[bibr55-00222429211047822] Yoffie David B . (1996), “Competing in the Age of Digital Convergence,” California Management Review, 38 (4), 31–53.

