# Supplementary material for: Attribute Embedding: Learning Hierarchical Representations of Product Attributes from Consumer Reviews
Source: J Mark. 2021 Nov 17;86(6):155–75. doi: 10.1177/00222429211047822 (PMC13038142; doi:10.1177/00222429211047822)
Supplement: sj-pdf-1-jmx-10.1177_00222429211047822 - Supplemental material for Attribute Embedding: Learning Hierarchical Representations of Product Attributes from Consumer Reviews [file sj-pdf-1-jmx-10.1177_00222429211047822.pdf]

Attribute Embedding: Learning Hierarchical Representations of Product Attributes from Consumer Reviews

Xin (Shane) Wang (xwang@ivey.uwo.ca), Jiaxiu He (hejiaxiu@gmail.com),
David Curry (david.curry@uc.edu), Jun Hyun (Joseph) Ryoo (jhryoo@cityu.edu.hk)

Web Appendix

Web Appendix A: Models and Equations Used in this Research	2
Web Appendix B: Data Preparation	4
Web Appendix C: The Seven Cluster Solution for Meta-attributes	8
Web Appendix D: Sentiment Analysis	10
Web Appendix E: Web-Based Surveys to Evaluate Meta-attributes	16
Web Appendix F: Product-Series-Level Market Structure	23
Web Appendix G: Product Positioning Maps Using Combinations of Published Techniques	25
References	27

Disclosure: These materials have been supplied by the authors to aid in the understanding of their paper. The AMA is sharing these materials at the request of the authors.

WEB APPENDIX A: MODELS AND EQUATIONS USED IN THIS RESEARCH

Model		Equation	Notation & Purpose
Skip gram	(1)	$\frac{1}{\lvert V\rvert}\sum_{t=1}^{\lvert V\rvert}\sum_{-k\le j\le k,j\ne 0}\log p(w_{t+j}\,\lvert w_t),$	w : product attribute (a noun or noun phrase)<br>v_w : a d-dimensional semantic vector<br>k : the "window size" of the context<br>w_t : a phrase at location t<br>$\lvert V\rvert$: size of the entire vocabulary<br>V : corpus<br>Attribute embedding: maximize the log probability
	(2)	$\arg\max_\theta \prod_{w\in V}\prod_{c\in c(w)} p(c\lvert w;\theta) =$<br>$\arg\max_\theta \sum_{\text{All }(w,c)}\log p(c\lvert w;\theta)$	$c\in c(w)$: the set of all contexts for attribute w<br>θ : parameter vector θ to maximize the corpus probability, θ consists of v_w's in $\mathbb{R}^d$ for $w\in V$, and v_c's in $\mathbb{R}^d$ for $c\in c(w)$<br>$\lvert C\rvert\times\lvert V\rvert\times d$: Total parameters
	(3)	$p(c\lvert w;\theta)=\frac{\exp(v_c^{\mathrm{T}}v_w)}{\sum_{c'\in C}\exp(v_{c'}^{\mathrm{T}}v_w)}.$	Multinomial logit model trained using the v_w as the independent variables to predict the conditional probability of the context given the attribute.
Negative Sampling	(4)	$=\log\exp(v_c^{\mathrm{T}}v_w)-\log\sum_{c'\in C}\exp(v_{c'}^{\mathrm{T}}v_w)$	Alternative expression for $\log p(c\lvert w;\theta)$ in (2).
	(5)	$\log\frac{1}{1+\exp(-v_c^{\mathrm{T}}v_w)}+\sum_{i=1}^{n}\log\frac{1}{1+\exp(v_{c_i'}^{\mathrm{T}}v_w)}$	Expression to replace (4) in (2).<br>c_i's : *n* negative samples randomly generated from a "noise distribution"
Pairwise similarity	(6)	$\frac{v_{w1}^{\mathrm{T}}v_{w2}}{\lVert v_{w1}\rVert\lVert v_{w2}\rVert},$	v_{w1},v_{w2} : semantic vectors representing two attributes
Singular Value Decomposition	(7)	$R=U\Sigma V^T$	A SVD expresses the m by n matrix R as the product of three matrices; U an $m\times d$ *product attribute* by *usage situation* orthogonal matrix, V an $n\times d$ *linguistic context* by *usage situation* orthogonal matrix, and Σ a $d\times d$ diagonal matrix of weights.
Sentiment Analysis	(8)	Sentence polarity index:<br>$\frac{P_{ij}-N_{ij}}{T_{ij}}$:	MA_{ij} : meta-attribute *j* – a bundle of lower-order attributes – for brand i.<br>P_{ij} : the number of review sentences with positive sentiment<br>N_{ij} : the number of review sentences with negative sentiment<br>T_{ij} the total number of sentences that contain meta-attribute *j* in a brand *i* as the sentiment score of MA_{ij}

			Find the subset of all review sentences that have high semantic similarity with the lowest-order product attributes contained in MA_{ij}. Feed these sentences to a sentiment classifier trained by labeling *polarity* in a subset of sentences.
Validation	(9)	$Accuracy = (truepos + trueneg)/(allpos + allneg)$<br>$Precision = truepos/(truepos + falsepos)$<br>$Recall = truepos/(truepos + falseneg)$<br>$F1 = (prec * recall/prec + recall)$	**Confusion Matrix** Actual<br><table><tr><td></td><td></td><td>POSITIVE</td><td>NOT POS</td></tr><tr><td rowspan="2">Predicted</td><td>POSITIVE</td><td>*truepos*</td><td>*falsepos*</td></tr><tr><td>NOT POS</td><td>*falseneg*</td><td>*trueneg*</td></tr></table>

WEB APPENDIX B: DATA PREPARATION

Collection and Preprocessing of Tablet Product Reviews

We used the software published by Wang, Mai, and Chiang (2014) to collect online product reviews used in the empirical study. The web crawler downloaded product reviews under the *Tablets & Tablet PCs* category from Amazon.com (hereafter Amazon) and removed the HTML tags. In addition to the free-form text reviews, we downloaded the star rating, review date, and general product information such as brand and product name. This information was stored in an SQLite database. The preprocessing procedure also identified each product review's linguistic components using three steps.

1. *Tokenization*, to separate words and detect sentence boundaries. Tokenization breaks reviews into sequences of elementary units, such as individual words, sentences, and punctuation marks. Simultaneous *lemmatization* transforms words to their root forms to remove differences in form that alter meaning only slightly. For example, *computers* and *computer's* become *computer*, and *are, am,* and *is* become *be*.
2. *Part-of-speech (POS) tagging*, to identify words by their parts of speech, such as adjectives, nouns, noun phrases, verbs, or verb phrases. We used the Penn Treebank Tags (Marcus, Marcinkiewicz, and Santorini 1993) for POS representation. For example, the sentence, "The handwriting recognition is fantastic," would be tagged: "The (DT) handwriting (NN) recognition (NN) is (VBZ) fantastic (JJ)," where DT stands for determiner, NN stands for noun, VBZ indicates a third-person singular verb, and JJ denotes adjective.
3. *Common bi-gram and noun-phrases identification*. Words that appear frequently together in sequence are identified using the methods proposed by Mikolov et al. (2013b).

Several open-source natural language processing (NLP) packages are available to handle these preprocessing tasks. Our Python implementation relies on open-source libraries, including NLTK (Bird 2006), TextBlob, and Gensim (Řehůřek and Sojka 2010).

Product Attribute Extraction

Integrating NLP techniques enables us to infer product attributes. Previous research has investigated various techniques to elicit product attributes automatically. Unsupervised learning approaches are preferable (Wei et al. 2009), because they do not require annotated review sentences for training purposes. The framework proposed by Hu and Liu (2004), which employs association rule mining (Agrawal and Srikant 1994) to identify frequent noun phrases from reviews, has proven valid (Archak, Ghose, and Ipeirotis 2011). We expand their framework by introducing a sequence of filtering and pruning techniques to extract noun phrases from product reviews as candidate product attributes.

Our method starts by extracting noun phrases that include fewer than some predefined number of words (e.g., 3) from review sentences. Stop words such as *I*, *the*, *was*, and *a* are filtered out of each noun phrase. The most frequently mentioned noun phrases become candidates for product attributes. These candidates typically exhibit three key problems that we resolve in subsequent steps. Candidates can contain:

1. redundant nouns that are parts of other noun phrases. For example, *life* is a redundant noun in the noun phrase *battery life*.
2. noun phrases that are not specific to the product category of interest (e.g., tablets), such as *something*, *people*, *fact*, *others*, or *today*.
3. noun phrases that are brand names or general product categories, such as *iPad*, *Samsung*, *tablet*, and *tablet computer*.

Our method adopts the redundancy pruning procedure proposed by Hu and Liu (2004) to solve the second problem, which computes a measure of how often a phrase appears alone, rather than as part of another phrase. The percentage of reviews that contain a phrase constitute support for the phrase, and the support of a phrase appearing alone (but not as a part of another phrase) is defined as *pure support*. For example, pure support for the term *life* is the percentage of reviews that contain *life* as a noun phrase but

no supersets (e.g., *battery life*) of that phrase. When a phrase's pure support is lower than its support, the phrase by itself carries less meaning in that context. Therefore, we retain only noun phrases with a pure-support to support ratio greater than a threshold. Although in Hu and Liu's (2004) original definition, pure support cannot be negative, we employed a heuristic to achieve faster computation that can result in negative values for pure support. In practice, this variation does not affect results.

To filter out common and irrelevant noun phrases, we apply a likelihood ratio test (Yi et al. 2003). For each candidate noun phrase, our method computes relative frequency discrepancies between reviews of the product category of interest (e.g., tablets) and reviews of an irrelevant product category, such as books. We use reviews from a different product category, instead of a more general corpus, because many noun phrases are specific to e-commerce (e.g., *shipping, Amazon*) but do not refer to the product, so they should not display substantial frequencies in the reference model. Noun phrases with high likelihood ratios are candidate product attributes for the product category of interest. Phrases with likelihood ratios below a threshold are considered irrelevant and are eliminated. For example, "touchscreen" should appear frequently in tablet reviews but not in book reviews, so it would produce a high likelihood ratio. Mathematically, the likelihood ratio $-2\log\lambda$ is defined as: $-2\log\lambda = \begin{cases} -2\,lr & if\ r_2 < r_1 \\ 0 & if\ r_2 \geq r_1 \end{cases}$, where;

$lr = (C_{11} + C_{21})\log r + (C_{12} + C_{22})\log(1 - r) - C_{11}\log r_1 - C_{12}\log(1 - r_1) - C_{21}\log r_2 - C_{22}\log(1 - r_2),$

where; $r_1 = \frac{C_{11}}{C_{11}+C_{12}}$, $r_2 = \frac{C_{21}}{C_{21}+C_{22}}$, $r = \frac{C_{11}+C_{21}}{C_{11}+C_{12}+C_{21}+C_{22}} r = \frac{C_{11}+C_{21}}{C_{11}+C_{12}+C_{21}+C_{22}}$ and C_{11}, C_{12} are counts of relevant product reviews that contain and do not contain the phrase, respectively, whereas C_{11}, C_{12} are counts of irrelevant texts that contain and do not contain the phrase, respectively. Although the likelihood ratio is asymptotically distributed as χ^2, in practice the filtering threshold is set much higher than the traditional $p = .05$ level.

For the third problem, we manually filter out a set of unrelated noun phrases, such as brand and product names, and remove them from the list. Table WB.1 provides examples of noun phrases and their related linguistic measures to illustrate the method for extracting product attributes. In our case, we set the

pure support-to-support ratio threshold to .1, the likelihood ratio cutoff to 2,000, and a support threshold of .00385 (i.e., .385% of the reviews mentioned these attributes). These thresholds may be adjusted subjectively, depending on the size of the data set and how exhaustive the researcher wants.

Table WB.1: Examples of Product Attribute Extraction and Filtering

**Noun Phrases**	**Support**	**Pure Support**	**Pure Support/Support Ratio**	**Likelihood Ratio**	**Candidate Attribute?**
Tablet	.459	.0881	.193	41053	No
One	.272	.0644	.236	20.2	No
Apps	.21	.0356	.169	12526	Yes
Time	.172	.0624	.3634	42.9	No
Battery Life	.1	.1	1	—	Yes
WiFi	.1	.034	.344	4948	Yes
Camera	.0792	.0295	.372	3263	Yes
Market	.0692	-.0398	-.5759	1748	No
Life	.02426	-.0757	-4.104	8259	No

WEB APPENDIX C: THE SEVEN CLUSTER SOLUTION FOR META-ATTRIBUTES

After applying the attribute embedding model and computing cosine similarities between attributes, we use hierarchical clustering to construct the attribute hierarchy. We adhere to suggestions by Punj and Stewart (1983) to address four issues: data transformations, solution, validity, and variable selection. To cluster attributes, we applied Ward's minimum variance linkage hierarchical clustering procedure, which produces a nested sequence of partitions with an all-inclusive cluster at the top and individual product attributes at the bottom (Ward 1963). Unlike k-means or k-centroid, clustering, hierarchical clustering does not presume a particular number of clusters. To examine the validity of results, we first examine qualitatively whether the results are meaningful and useful (Punj and Stewart 1983), then assess results quantitatively.

Qualitative Assessment: As we show in the main text, the meta-attributes represented by the seven-cluster solution reflect meaningful differentiations in usage situations. Results from our consumer survey and from comparisons to other methods show meaningful convergence.

Quantitative Assessment: Figure WC.1 shows that according to the internal validation criteria – the average Silhouette coefficient and the Dunn index – the seven-cluster scenario (i.e., seven meta-attributes) is reasonable for our empirical study. The average Silhouette coefficient (Rousseeuw 1987) combines measures of both cohesion and separation for observations in a cluster, defined as $\frac{(b_t - a_t)}{max(a_t, b_t)}$, where a_t is the average distance from observation *t* to the other points in the cluster, and b_t is the minimum average distance from *t* to the clusters that do not contain this observation. The Dunn index (Dunn 1974) is another measure to identify compact, well-separated clusters, using the ratio of the smallest distance between observations from different clusters to the largest distance between observations from the same cluster. For both measures, larger values are desirable. Figure WC.1 shows that the Dunn index achieves its second highest value at 7. The maximum value is at 2 but the meta-attributes at this level are too coarse to be cleanly interpreted, plus the average Silhouette index is higher at 7 than at 2 and flattens out after 7.

Figure WC.1: Internal Measures of Cluster Validity

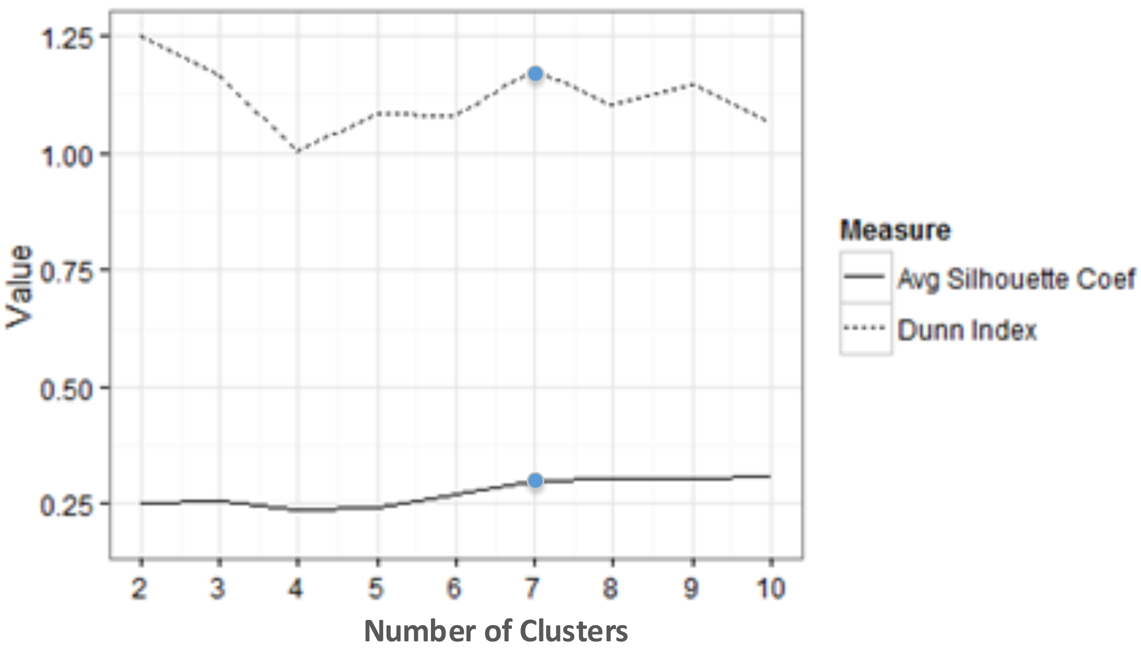

Robustness: Lastly, we checked the robustness of the seven-meta-attribute solution vs. four- and ten-attribute solutions to regenerate our static positioning map. We computed the QAP correlations of the resulting distance matrices with those from our original solution. QAP computes correlations between entries of two square matrices to test their association over possible permutations of the rows and columns of each matrix. It involves two steps: (1) compute Pearson's correlation coefficient (or other association measure; e.g., simple matching, Jaccard, Goodman-Kruskal Gamma and Hamming distance) between corresponding cells of the two data matrices, and then (2) randomly permute rows and columns (synchronously) of one matrix then recompute the correlation (or other measure). Our QAP tests yield 0.879 for the four meta-attribute solutions and 0.886 for the ten meta-attribute solution ($p < 0.01$ in each case). Thus, although the optimal solution uses seven meta-attributes, results are similar when using substantially coarser-grained or finer-grained ontologies.

WEB APPENDIX D: SENTIMENT ANALYSIS

Sentiment Analysis: Method

Sentiment analysis diagnoses natural language text to reveal consumers' positive or negative feelings about product attributes. To train our sentiment classifiers, we randomly selected a set of 2,000 review sentences and had two coders manually label each sentence as positive, negative, or neutral. In the training set, 37.6% of sentences were classified as positive, 43.1% as negative, and 19.4% as neutral. The labeled set was used to train two binary classifiers, one to detect positive and one to detect negative opinions, for each of five machine learning algorithms. This approach is known as the "one-versus-all" scheme and is extremely powerful (Rifkin and Klautau 2004). The five classifiers we compared are: Maximum Entropy, Decision Trees, Naïve Bayes, Random Forests, and the Support Vector Machine. We used the following predictor variables with each classifier.

1. *Raw sentiment scores according to the general lexicon*. A list of positive and negative opinion words determines the score (Blair-Goldensohn et al. 2008; Hu and Liu 2004). For example, words such as *great*, *fantastic*, and *thrilled* are positive opinion words, and words such as *damaged*, *flawed*, and *weak* are negative opinion words. A dictionary of around 6800 words, http://www.cs.uic.edu/~liub/FBS/opinion-lexicon-English.rar, informed our empirical study. If a sentence has *n* positive and *m* negative words, its raw sentiment score is $(n-m)$.
2. *Magnitude of opinion*. This measure is calculated as an indicator of how strong the opinion is: $Magnitude = \frac{(n-m)}{(n+m)}$. When magnitude is large in absolute value, the opinion of the sentence is highly polarized.
3. *Star Rating*. The star rating of the product provided by the reviewer. If the star rating is low, each sentence in the review is more likely to express a negative opinion.
4. *Domain-specific textual features*. Textual features were selected in two steps: the top 200 words with substantive meaning were selected using an *importance index* (Eliashberg, Hui, and Zhang 2007). Words that appear in almost every review and words that appear in very few reviews are screened out by the importance index. The index in our study is calculated using $I_i = \left(1 - \frac{\sqrt{d_i}}{D}\right)\sqrt{N_i}$. Here, d_i is the number of reviews containing the ith word; D is total number of reviews, and N_i is the total frequency of this word.

To compare the five algorithms, we followed protocols suggested by Feldman and Sanger (2007) and Hastie, Tibshirani, and Friedman (2009).

Descriptions of Classifiers

We use S to denote the sentiment of a sentence, d_t to indicate a sentence in the training set, d_i for a sentence with an unknown sentiment label, and $\boldsymbol{x}_i = (x_1, x_2, \ldots, x_n)$ as a feature vector for sentence i.

Maximum Entropy: The maximum entropy (ME) classifier estimates the posterior probability distribution over classes using linear functions of the input features. The ME principle is to choose a model consistent with all facts but otherwise as uniform as possible (Berger, Pietra, and Pietra 1996). For a binary response variable, the ME classifier is commonly known as a *logistic regression classifier*, which is a generalized linear model. The model specifies a log-odds (logit) transformation of the response using the form, $log\frac{Pr(S=1|d_i)}{Pr(S=0|d_i)} = \beta_0 + \boldsymbol{\beta}_1^T \boldsymbol{x}_i$ (Hastie, Tibshirani, and Friedman 2009). For example, in our study, an event $S = 1$ indicates that a sentence contains a positive sentiment, and $S = 0$ suggests that the sentence contains no positive sentiment. Each entry in $\boldsymbol{\beta}$ can be interpreted as the weight for the corresponding feature. The parameter vector $\boldsymbol{\beta}$ is estimated using optimization algorithms such as iterative scaling, conjugate gradient, or the BFGS algorithm (Malouf 2002).

Decision Tree: A classification tree or decision tree (DT) classifier builds a tree-structured flowchart to select the response class for a given set of input values. Starting from the root node, at each node of the tree, the algorithm checks a logical condition for one input feature and selects a child branch. On reaching a leaf or terminal node, a classification label is assigned. For the construction of the decision tree, a feature gets chosen at each step, and the decision of where to split ensures maximal information gain (Bird, Klein, and Loper 2009).

Naïve Bayes: A naïve Bayes (NB) classifier is based on the assumption that each feature is conditionally independent from all others. Although this assumption is obviously not true, the NB

classifier works well for text classification. According to Bayes rule, the probability that a sentence belongs to a class is:

$$Pr(S = s|d_i) = \frac{Pr(d_i|S=s)\,Pr(S=s)}{Pr(d_i)} \quad \text{(W1)}$$

If the independence assumption holds, the most probable category for a sentence d_t is calculated as:

$$S^* = argmax_{s \in S}\, Pr(S = s) \prod_{j=1}^{n} Pr(x_j|s) \quad \text{(W2)}$$

Training a NB classifier requires estimating each probability. Counting the number of positive sentences in the training set, divided by the total number of sentences in the training set, yields the maximum likelihood estimate of $Pr(S \equiv positive)$. The probability of a feature, given a class label $Pr(x_j|s)$ is estimated from the training set using Laplace smoothing:

$$Pr(x_j|s) = \frac{1+\sum_{d_t \in S} I(x_j, d_t)}{n+\sum_{j=1}^{n} \sum_{d_t \in S} I(x_j, d_t)} \quad \text{(W3)}$$

where $I(x_j, d_t)$ is 1 if feature x_j occurs in sentence d_t, and 0 otherwise. These equations assume that the features are binary. Non-binary features, such as user ratings, can be converted into binary features by binning or replacing $Pr(x_j|s)$ with the estimated normal density for each class (John and Langley 1995).

Random Forests: Individual decision trees tend to overfit data. The Random Forest (RF) idea was first proposed by Ho (1995) and then extended by Breiman (2001). RF constructs a large number of decision trees in training for classification and regression. Classification is based on either the mode or mean prediction from the resulting ensemble of individual trees. The approach essentially integrates aggregation (bagging) and bootstrapping (sub-sampling) directly into the "fitting" stage in order to reduce overfitting and improve out-of-sample forecasts relative to those from a single DT classifier. The RF method randomly sub-samples observations from the total dataset and predictors from the predictor set. It also systematically sub-samples predictor variables at each node in a given decision tree by choosing the predictor variables that yield the best split at that node. Consequently, the method deals naturally with unbalanced and missing data. The steps in the RF algorithm are roughly as follows.

1. Split the total sample into hold-out and training sub-samples,

2. In the training sample

    2.1. Set the hyper-parameter $size\{T\}$; where $T \equiv set\ of\ decision\ trees$

        2.1.1. Sample N observations at random with replacement from the dataset

        2.1.2. At each node, by tree $t \in \{T\}$

            2.1.2.1. Select $m \ll p$ predictor variables at random from the set of all predictors *p*. In our work *p* = 4 and RF randomly selects 2 (=$\sqrt{4}$) features at each node of a split.

            2.1.2.2. Use the predictor variable $p^* \in m$ that maximizes predictive accuracy at that node

            2.1.2.3. GoTo 2.1.1.

            2.1.2.4. Stop once T is exhausted.

        2.1.3. Predict using either the mode or mean predictions from the ensemble of individual trees.

3. In the hold-out sample

    3.1. Assess the RF classifier using out-of-sample hit-rates and hit-rates relative to chance.

    3.2. See Web Appendix A, *accuracy*, *prediction*, *recall*, *F1*.

Support Vector Machine: Support vector machines (SVM) were proposed by Cortes and Vapnik (1995). They have become very popular as a classification method due to their scalability and performance. A SVM finds a separating hyper-plane between two classes. Importantly, a SVM's learning ability is independent of the dimensionality of the feature space (Joachims 1998). Therefore, this method is particularly well suited for text classification due to the high number of features usually contained in a text document. For details, see Burges (1998) and for applications in marketing, see Cui and Curry (2005).

Bagging

To increase prediction accuracy, we created a bagging predictor for each method except RF (Breiman 1996). (The RF method employs bagging (step 2.1.3) as an integral part.) In our empirical study, the training examples were bootstrapped over 15 rounds, and in each round, each separate classification model was fit to the data in each bootstrapped sample. For a given classification technique, we predict

new cases using the majority vote of the 15 rounds for that classifier. Bagging produces more stable prediction results and offers substantial gains in prediction accuracy (Breiman 1996; Hastie, Tibshirani, and Friedman 2009). The following pseudocode describes our algorithm:

1. Given labeled sentences (d_1, S_1), ..., (d_m, S_m) in a training set,
2. For training round t = 1, ..., T = *15*:
    a. Select (with replacement) *m* random examples from the training set.
    b. Train the classifier (e.g., SVM) in round (h_t) using each bootstrapped sample.
3. Classify new cases using the rule: $H(d_i)$ = majority ($h_1(d_i)$, ..., $h_T(d_i)$).

In rare cases (0.34% of our observations), the positive and negative classifiers give conflicting predictions. We used voting as the tiebreaker, i.e., the number of individual rounds inside the bagging procedure with positive versus negative predictions. For example, if 10 of the 15 rounds (for the positive classifier) predict positive and 8 of the 15 rounds (for the negative classifier) predict negative, the sentence is classified as positive. To train the classification tree and NB classifiers, we used the Python Natural Language Toolkit (Bird, Klein, and Loper 2009). To train the SVM and ME classifiers, we used the Scikit-learn dataset (Pedregosa et al. 2011). Both datasets are freely available under open-source licenses. Accuracy measures were estimated using 10-fold cross-validation by classifier.

Training Rounds

We used two rounds of comparisons because the Random Forest method includes bagging, hence is compared to the best classifier from round one.

Round 1 Results: Table WD.1 shows the performance of Maximum entropy, Classification tree, Naïve Bayes, and the SVM. The SVM exhibited the best overall "bagged" performance in these comparisons because it dominates on every comparison metric. The SVM achieves *precision* of .741 and *recall* of .685 for positive sentiments and *precision* of .727 and *recall* of .615 for negative sentiments. For example, of all the sentences that the classifier determined to be positive, 74.1% were also identified as positive by our raters. Conversely, of all the sentences that raters judged as expressing positive

sentiment towards an attribute, the classifier correctly detected 68.5%. These results are comparable to or better than those for sentence-level sentiment analyses in extant research (Gamon et al. 2005; Meena and Prabhakar 2007; Täckström and McDonald 2011).

Table WD.1: Round 1 Performance of Sentiment Classifiers

	**Positive**				**Negative**			
	Accuracy	Precision	Recall	F1	Accuracy	Precision	Recall	F1
Maximum entropy	.748	.716	.682	.699	.775	.709	.607	.654
Classification tree	.725	.728	.625	.673	.755	.691	.545	.609
Naïve Bayes	.738	.712	.675	.693	.744	.695	.587	.636
**SVM**	**.766**	**.741**	**.685**	**.712**	**.781**	**.727**	**.615**	**.666**

Round 2 Results: In round 2 we pitted the SVM directly against Random Forests. Table WD.2 shows results. For 7 of the 8 performance metrics there is no statistical difference between SVM and RF. However, the SVM statistically outperforms Random Forests on *recall* for positive classification ($p < 0.05$).

Table WD.2: Round 2: Head-to-Head Comparison: Random Forests vs. the Support Vector Machine

	**Positive**				**Negative**			
	Accuracy	Precision	Recall	F1	Accuracy	Precision	Recall	F1
SVM	0.728	0.714	0.621	0.664	0.739	0.683	0.544	0.606
RF	0.735	0.723	0.594	0.652	0.746	0.697	0.546	0.612
p-value	0.31	0.26	**0.04**	0.21	0.31	0.17	0.45	0.35

Selected Classifier for our Main Study

We used the Support Vector Machine for sentiment classification in our work in the main text for the following reasons. First, the two methods are statistically tied on 7 of 8 performance metrics, but the SVM statistically outperforms RFs on *recall*. Second, by definition, the RF method employs "bagging" and "bootstrapping" directly; whereas our SVM method only employs "bagging" post hoc. Third, although, in general randomly selecting a subset of features can address overfitting in training, in our application it leads to information loss. Finally, the link between predictors and outcomes is more transparent with the SVM. Because RF sub-samples DTs as well as predictors within tree and at each node, it is difficult to trace its accuracy back to any particular predictor.

WEB APPENDIX E: WEB-BASED SURVEYS TO EVALUATE META-ATTRIBUTES

Survey 1: We recruited n = 101 participants from Amazon MTurk platform who reside in the United States, have at least 95% approval from their previous tasks, and confirmed, via MTurk, that they were owners of tablet computers. The survey consisted of two tasks in the following order. In the first task, three meta-attributes were randomly selected for each participant, and all of their respective engineered attributes were shown in random order. We then asked the survey participants to write a three-paragraph review for the tablets that they owned, with each paragraph containing at least two sentences that together mention at least two of the engineered attributes shown. In Figure WE.1, we plot the frequency of the attributes chosen to be reviewed by survey participants, with the overall average (8.66) indicated by the orange line. It is apparent that although each participant was randomly assigned three meta-attributes, some engineered attributes were chosen to be reviewed more frequently than others, likely due to the domain expertise required to recognize and review the less commonly known attributes.

Figure WE.1: Frequency of Attributes Reviewed by Survey Participants

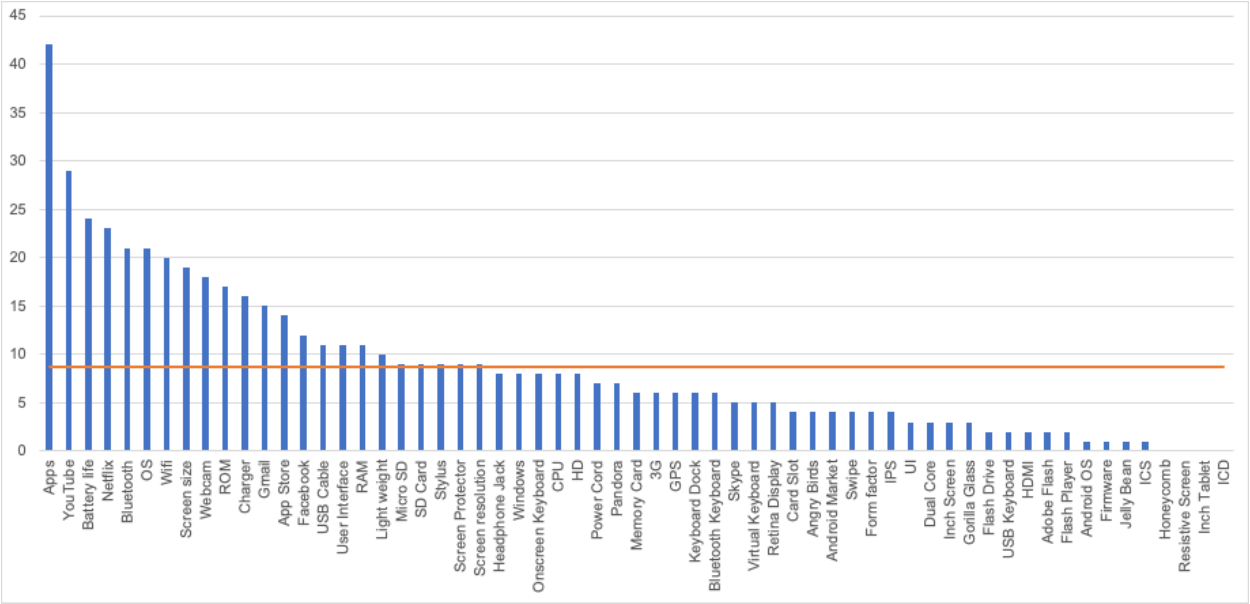

We assume that engineered attributes reviewed in the same paragraph by the survey participants are more likely to share implicit relationships for the reviewer than attributes that appear in separate paragraphs. After removing stop words, we calculate the correlations between the remaining words using

the phi coefficient. The phi coefficient measures correlation between binary variables—in our context, whether words are present in the same paragraph or not. Specifically, the phi coefficient for words *a* and *b* is calculated using the following equation:

$$\emptyset = \frac{n_{11}n_{00} - n_{10}n_{01}}{\sqrt{n_{a1} \times n_{a0} \times n_{b0} \times n_{b1}}} \qquad \text{(W4)}$$

where n_{11} is the number of paragraphs that contain both words *a* and *b,* n_{00} is the number of paragraphs that do not contain words *a* or *b,* and n_{10} and n_{01} are the number of paragraphs that contain only word *a* and only word *b,* respectively. n_{a1} (n_{b1}) is the total number of paragraphs that contain word *a* (*b*)*,* and n_{a0} (n_{b0}) is the total number of paragraphs that do not contain word *a* (*b*)*.* After calculating the phi coefficients, we plot the correlations greater than 0.20 as a network in Figure WE.2.

Figure WE.2: Network Plot of Attribute Correlations

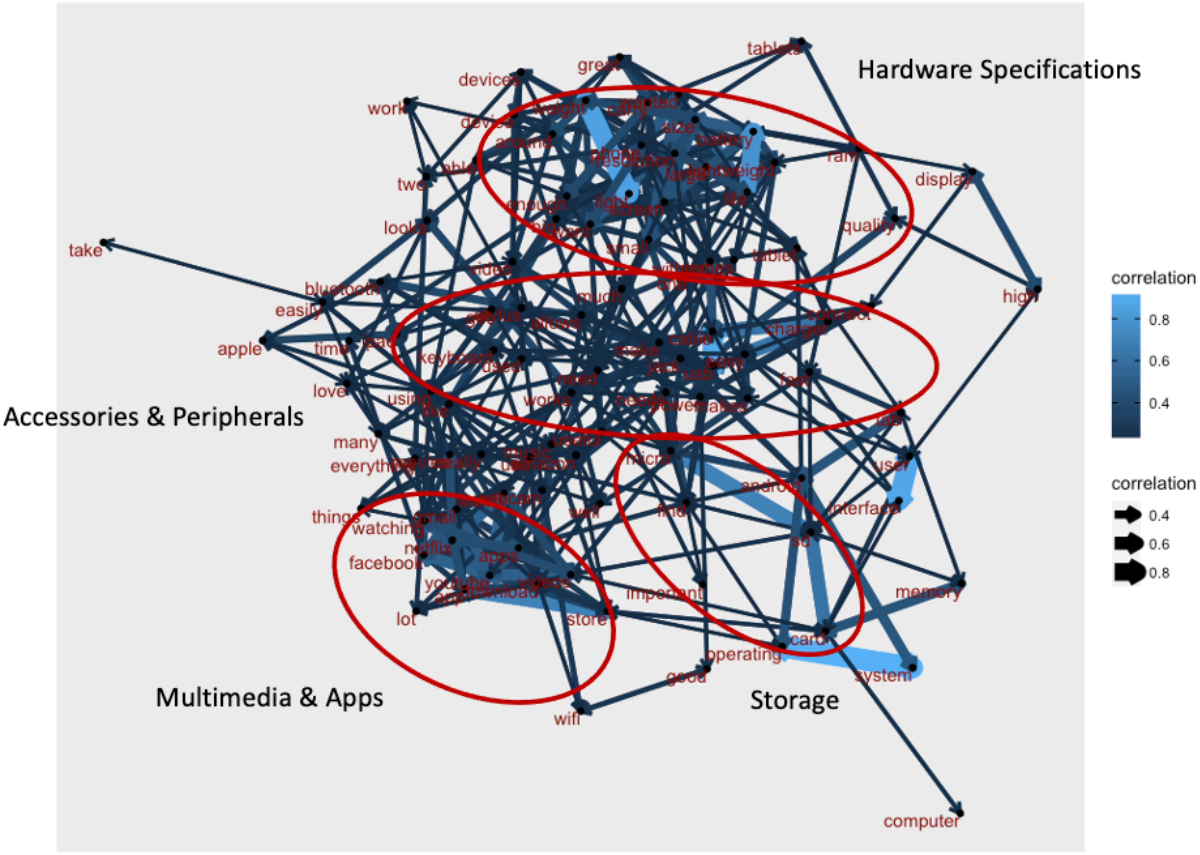

Table WE.1: Identified Engineered Attributes and Meta-Attributes in the Network

**Meta-Attributes**	**Clusters of Engineered Attributes**
Hardware Specifications	RAM, lightweight, battery, life, screen, size, weight, big, small
Accessories & Peripherals	stylus, USB, cable, charger, keyboard, cable, connect, jack
Storage	micro, SD, card
Multimedia & Apps	Gmail, Facebook, Netflix, YouTube, apps, store, videos, watching, download

Figure WE.2 reveals four clusters of engineered attributes (circled in red) that resemble the four meta-attributes found in our attribute hierarchy (see Table WE.1): Hardware Specifications, Accessories & Peripherals, Storage, and Multimedia & Apps. Attributes within these clusters are more likely to be reviewed in the same paragraph. The engineered attributes associated with the excluded three meta-attributes did not receive enough mentions from survey participants (Figure WE.1) to form clear and independent clusters.

In the second task of the survey, we presented participants the names of all seven meta-attributes in our attribute hierarchy. We then asked the participants to freely list three concrete features or specifications of tablets that came to their mind for each of the meta-attributes. Figure WE.3 shows the word clouds for each of the meta-attributes, generated using word frequency in the aggregated responses.

Figure WE.3: Word Clouds of Attributes in Survey Responses

Hardware Specification

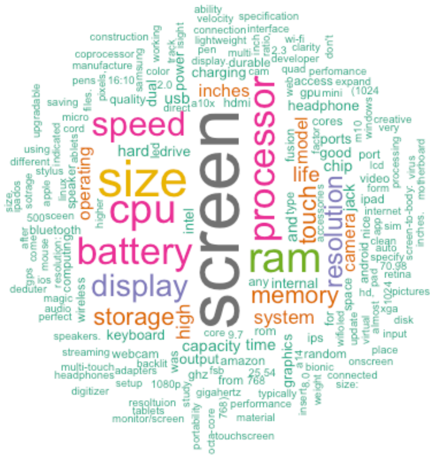

Storage

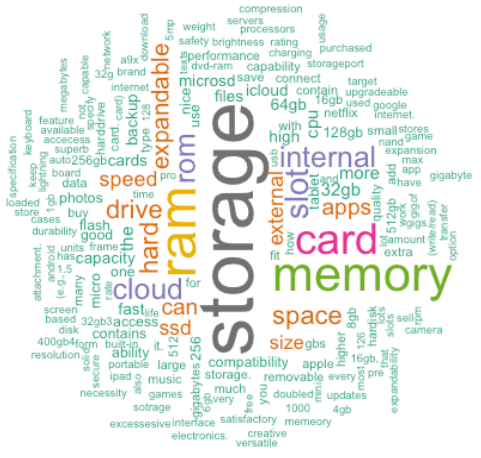

Wireless Connectivity

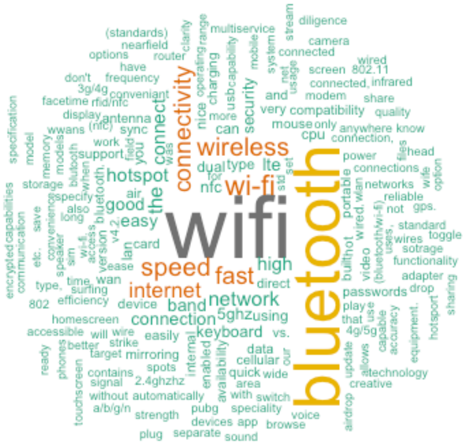

Accessories & Peripherals

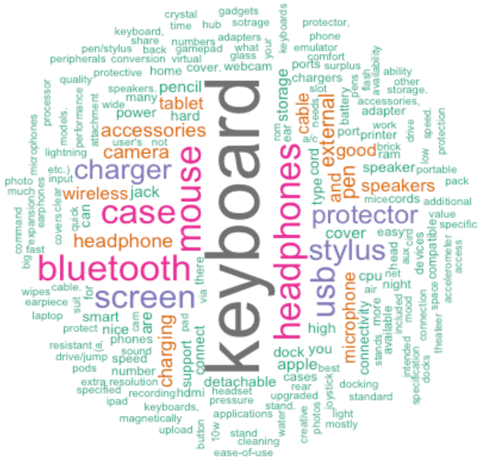

Multimedia & Apps

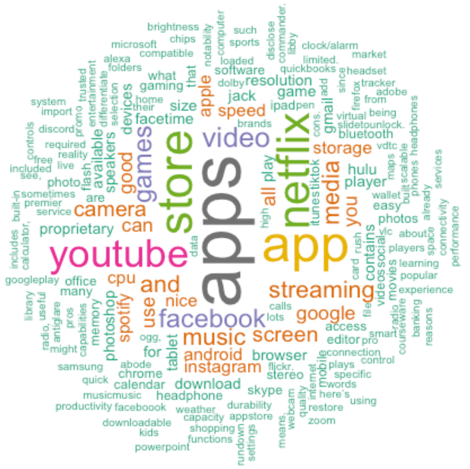

Operating System

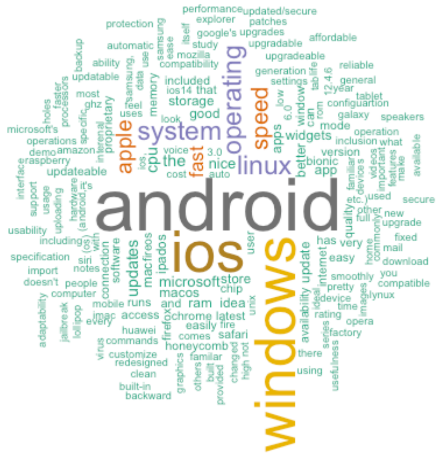

User Interface

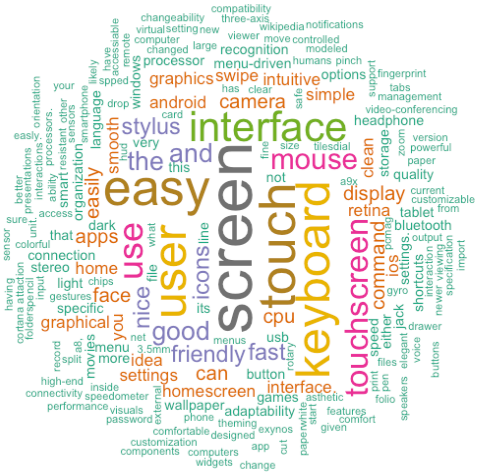

Qualitatively examining the word clouds, we find that the most frequently mentioned attributes, as indicated by their size and color in the word clouds, either match or are synonymous with the engineered attributes from our attribute hierarchy, providing face validity to our method. Examining the survey responses, we also note that each meta-attribute contains numerous less-frequently mentioned attributes, as indicated by their teal color in the word clouds. This highlights the drawback of using survey-based methods to elicit engineered attributes, as it is difficult to determine which of the less frequent attributes are informative. Since our attribute embedding model captures the contexts of how attributes are used in reviews, information from less frequently used words is incorporated in clustering and sentiment analysis.

Survey 2: We recruited n = 201 U.S.-based participants from Amazon MTurk (n = 229 started; an 87.8% completion rate); all with a task completion rate of at least 95%. Each participant was shown a list of nine engineered attributes; three engineered attributes randomly selected from each of three randomly selected meta-attributes. Participants were instructed to categorize the engineered attributes into three unlabeled groups of the same size (i.e., "Group A", "Group B," and "Group C") when considering their similarities in benefits and tablet functionality. An example of the task is shown in Figure WE.4. The participant simply clicks and drags each engineered attribute listed on the left-hand side to one of the three unlabeled groups on the right-hand side.

Figure WE.4: An Example Task from the Online Survey

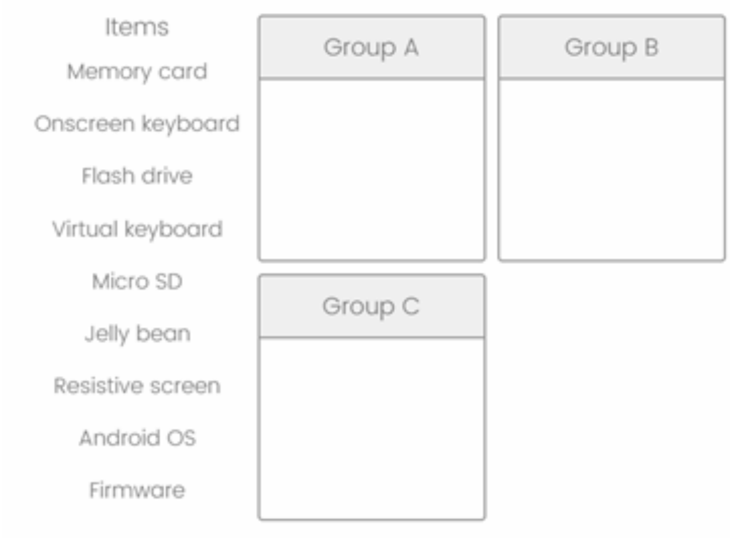

We then compared the survey responses to the meta-attributes from our attribute embedding model to calculate matching accuracy, defined as the ratio of correct number of engineered attributes over the total number of engineered attributes assigned for a specific meta-attribute. Since our focus is on the groupings of engineered attributes, accuracies with only a single correctly identified engineered attribute in their numerators were penalized to zero. We find an overall accuracy of 78.8%, indicating an adequate level of agreement between human judgement and the results from our attribute embedding model.

Survey 3: Participants (n = 158) completed the experiment online via Amazon MTurk (n = 179 started; an 88.3% completion rate). Each participant considered three randomly selected meta-attributes along with ten randomly selected engineered attributes. We employed a balanced, fractional factorial design to generate {meta-attribute, engineered attribute}-pairs shown to each participant. For example, a participant was equally likely to see the pair of {*MicroSD*, Storage} vs. *MicroSD* paired with any of the other six meta-attributes. Participants evaluated how well a given engineered attribute corresponded to each of the provided meta-attributes on a six-point scale ranging from 0 to 5, where 0 represents no correspondence at all and 5 indicates full correspondence. The survey also included instructions to help participants understand the question. Figure WE.5 shows an example of the task.

Figure WE.5: Task Instructions for the Online Survey

You will be asked a few questions about tablet computers on the next page.
The following example is designed to help you understand the question.
It is not intended to suggest answers.

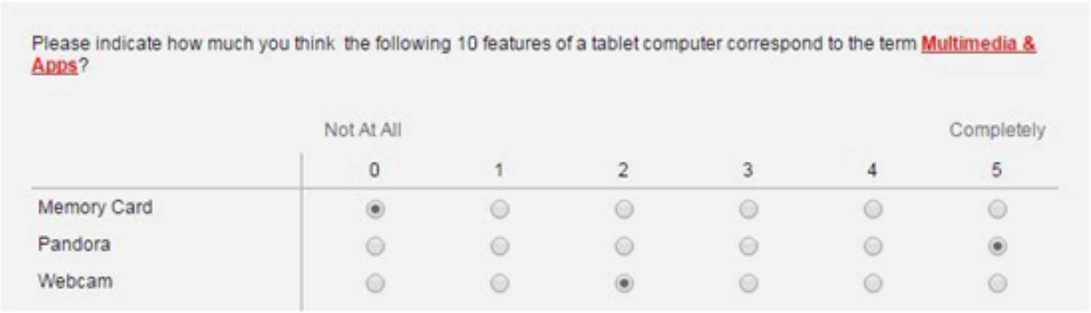

Memory Card	:	I do not see any relationship between **Memory Card** and Multimedia & Apps, so I chose "0; Not At All" as my answer.
Pandora	:	**Pandora** is definitely related to the concept Multimedia & Apps, so I chose "5; Completely" as my answer.
Webcam	:	To me **Webcam** is related to Multimedia & Apps, but the relationship is not that strong, so I chose "2" to indicate my measure of the relationship.

Table WE.2 summarizes the percentage of ratings greater than or equal to 3 for each meta-attribute and for engineered attributes contained in that meta-attribute (left panel, column convergence) and not contained in that meta-attribute (left panel, column discrimination). The right panel reports the average ratings. Results show that consumers view the correspondence in a manner consistent with the attribute hierarchy from our embedding model. The only meta-attribute with convergence below 70% is wireless connectivity; a category to which some human raters do not feel that "webcam" and "GPS" belong.

Table WE.2: Empirical Evaluation Results of Seven Meta-Attributes

**Meta-Attributes**	**Proportion of attributes rated > 3**		**Average rating**	
	**Convergence**	**Discrimination**	**Convergence**	**Discrimination**
Storage	100.0%	3.6%	3.944	1.120
Wireless Connectivity	60.0%	.0%	3.466	1.129
Tablet Accessories & Peripherals	90.0%	15.4%	3.881	2.070
Multimedia & Apps	91.6%	4.0%	3.922	1.772
Operating System	71.4%	3.6%	3.623	1.425
User Interface	85.7%	14.5%	3.682	2.157
Hardware Specifications	86.7%	9.1%	3.480	1.736

Note: *P*-values < 0.01 for all meta-attributes using a t-test for average ratings and a test of proportions for rating > 3. The null hypothesis for averages is that the mean rating from Convergence group is the same as the rating from the Discrimination group. The null hypothesis for proportions is that the percentage of the attribute falling in the meta-attributes (vs. not in) with the rating > 3 is equal.

WEB APPENDIX F: PRODUCT-SERIES-LEVEL MARKET STRUCTURE

Hierarchical sentiment analysis allows us to aggregate sentiment scores from the review level to various levels when conducting market structure analysis. We examined brand-level MSA in Figure 4. We now move to the level of product series to obtain deeper insights regarding how consumers perceive positions among a series of products manufactured by a given firm. A product series comprises similarly named SKUs that evolve over product generations to perfect the hallmark characteristics of the series (e.g., iPad 1, iPad 2, and iPad 3). All four brands that we previously analyzed had several series of tablets on the market by 2012. For this analysis, we aggregate sentiment scores to the product-series level for each of the seven meta-attributes, following the same procedures used for brand-level MSA. In Figure WF.1, the x-axis aligns with hardware including Hardware Specifications, Operating System, and Storage, while the y-axis aligns with functionality, including Wireless Connectivity, Accessories & Peripherals, Multimedia & Apps, and User Interface.

The product-series-level MDS yields insights beyond those at the brand level. First, Apple products are centered while competing products are positioned around them. This pattern reflects Apple's category leadership. Second, the three members of the iPad series are close to each other, indicating that consumers recognize their similarities. Consumers acknowledge the increased functionality from iPad 1 to iPad 2, which included front and back cameras. (Dual cameras enable consumers to use FaceTime to make video calls.[1]) But iPad 3 is not seen as functionally superior to iPad 2. iPad 3 introduced the Retina display (four times the pixels of iPad 2), 1080p back camera (vs. 720p; iPad 2), and the A5X processor (vs. A5; iPad 2). These changes are acknowledged as updated hardware components (x-axis) but do not constitute improved functionality (y-axis).[2] Third, the primary perceived differences among the series for the other three brands are horizontal, along the hardware dimension. This suggests that top vendors other than

---

[1] https://www.pocket-lint.com/tablets/news/apple/146888-history-of-the-apple-ipad, accessed June 3, 2020. FaceTime was launched in June 2010, two months after iPad was released.

[2] The biggest difference is the upgrade to the Retina display. However, as a CNET video report states, the improvement of video quality is rated from subtle to non-existence. See. https://www.cnet.com/videos/reasons-not-to-buy-the-new-ipad/, accessed June 3, 2020.

Apple were not likely to have a consistent marketing strategy during the early stages of the modern tablet era. Their strategies likely prioritized stacking up hardware components instead of enhancing functional benefits. Simply put, more sophisticated hardware – the trend of making a tablet more like a laptop – did not translate to "better" for consumers. Combined with the insights from our brand-level analysis, it suggests that while there exist opportunities to enhance consumer experience via innovations at the hardware level, it is perhaps more important to focus on the integration of software and hardware and to carry out a consistent marketing strategy over time.

Figure WF.1: Product Positions for Product Series using Consumer Sentiment

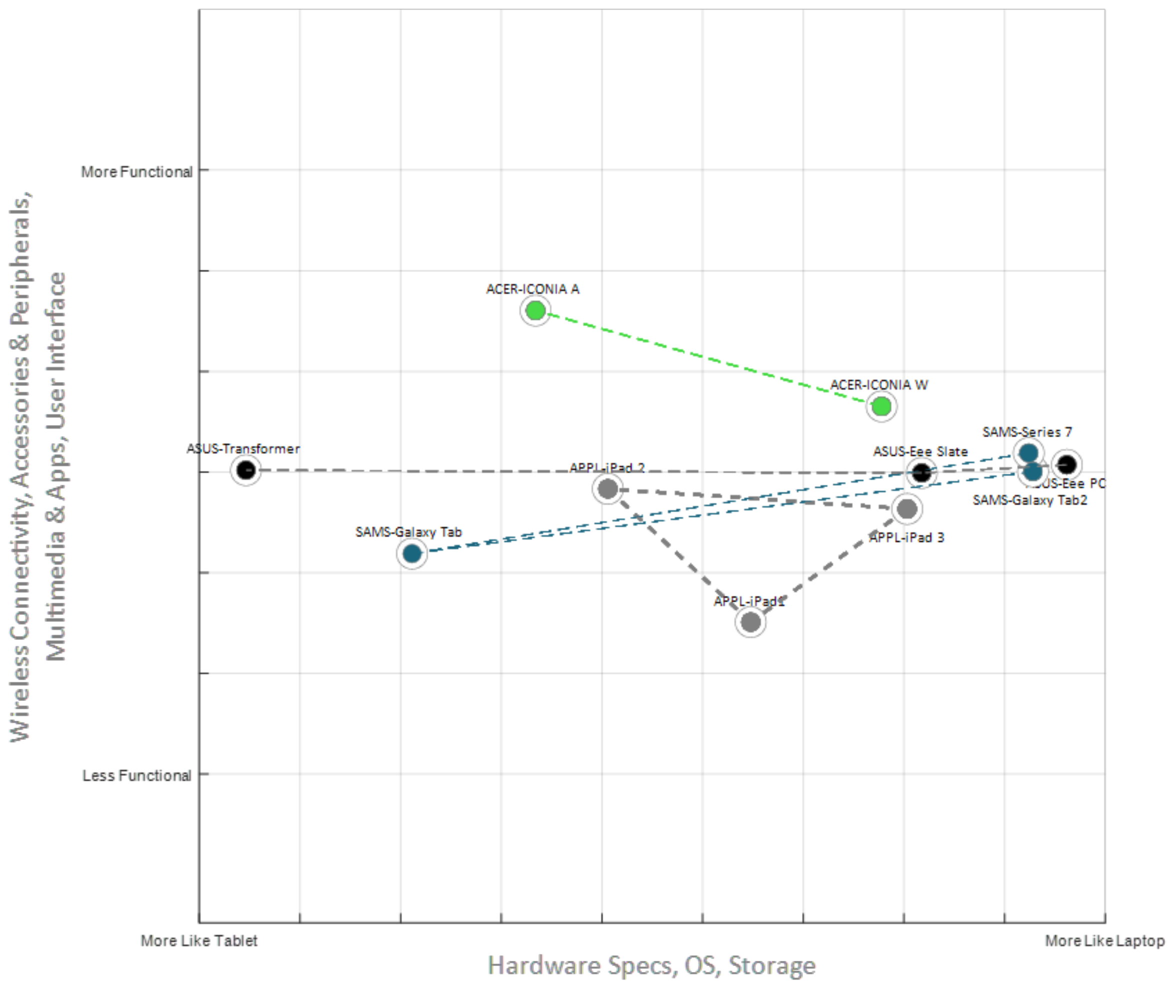

WEB APPENDIX G: PRODUCT POSITIONING MAPS USING COMBINATIONS OF PUBLISHED TECHNIQUES

Figure 4 in the main text shows our brand positioning map based on Euclidian distances between standardized brand-level sentiment scores. We reproduce Figure 4 in Figure WG.1a and compare our results to those obtained from alternative methods; including using engineered (vs. meta) attributes & sentiment analysis, meta-attributes & frequency counts, LDA & sentiment analysis, and brand co-occurrences. Results from these other methods are shown in their original units in Figure WG.1a and using identical axes limits in Figure WG.1b. (The figure is on the next page.) We compare these maps statistically on the basis of within cluster homogeneity, and qualitatively on the basis of interpretability.

As noted in the main text, our technique yields two distinct clusters and two outliers (Toshiba, HP). A visual scan of Figure WG.1b suggests that our clusters are more homogenous than those from the other methods. In addition, each of the other approaches has unique problems. Using *engineered attributes & sentiment analysis* the clusters are indistinct and the members of cluster A are highly dispersed. Using *meta-attributes and frequency counts* cluster A lacks homogeneity and the clusters overlap. However, cluster A is not as homogenous as with our method, Acer has drifted into cluster B (Figure WG.1a), and {HP and Toshiba} are not clearly distinguished as outliers. Finally, using *brand co-occurrences* fails to yield distinct clusters. The brands in cluster A are not as homogeneous as with our method.

Figure WG.1: Comparison of Positioning Maps

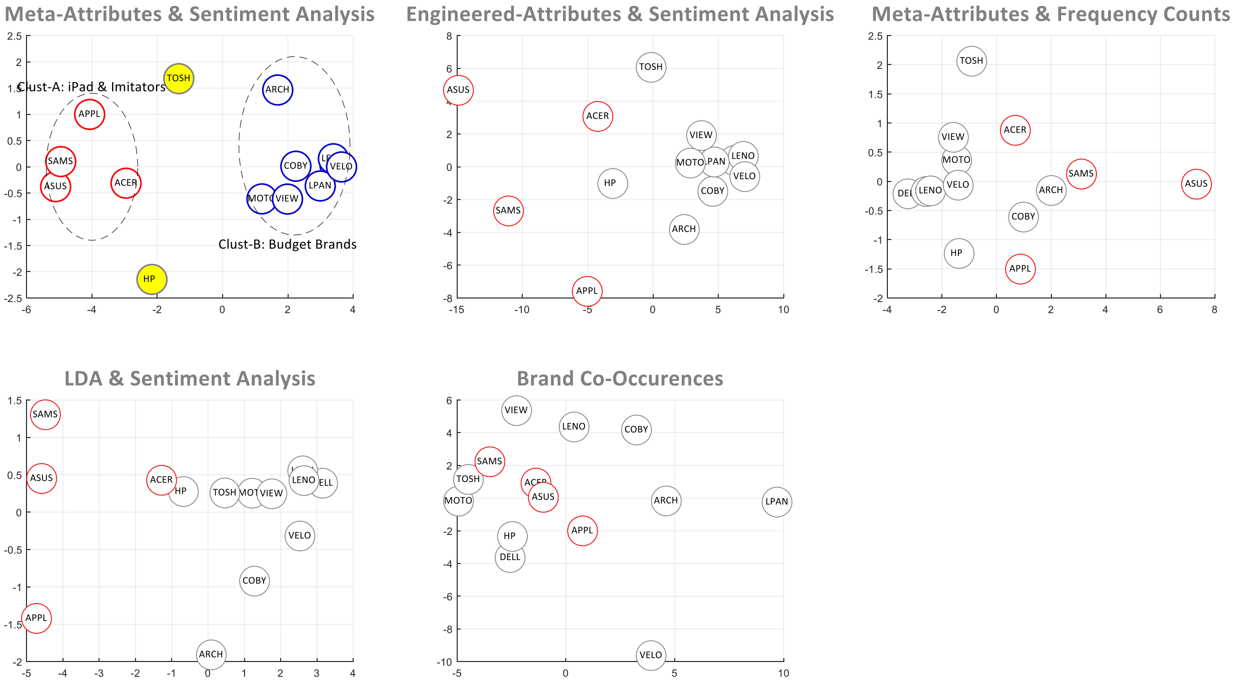

(a) Using Original Scaling

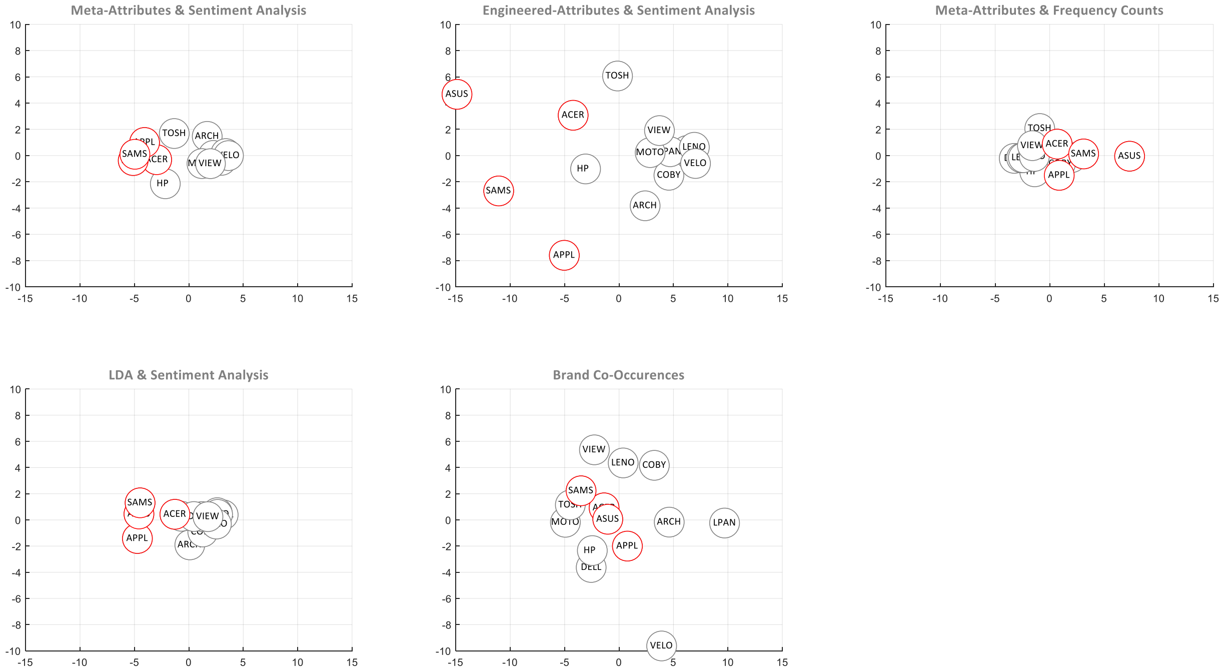

(b) Using Normalized Scaling

References

Agrawal, Rakesh and Ramakrishnan Srikant (1994), "Fast Algorithms for Mining Association Rules," *Proceedings of the 20th International Conference on Very Large Data Bases*, 487-499.

Berger, Adam L., Vincent J. Della Pietra, and Stephen A. Della Pietra (1996), "A Maximum Entropy Approach to Natural Language Processing," *Computational Linguistics*, 22 (1), 39-71.

Bird, Steven (2006), "NLTK: the Natural Language Toolkit," *Proceedings of the COLING/ACL on Interactive presentation sessions*, 69-72.

Bird, Steven, Ewan Klein, and Edward Loper (2009). *Natural Language Processing with Python*, O'Reilly Media Inc.

Blair-Goldensohn, Sasha, Kerry Hannan, Ryan McDonald, Tyler Neylon, George A. Reis, and Jeff Reynar (2008), "Building A Sentiment Summarizer for Local Service Reviews," *WWW Workshop on NLP Challenges in the Information Explosion Era (NLPIX)*.

Breiman, Leo (2001), "Random Forests," *Machine Learning*, 45, 5–32.

Burges, Christopher (1998), "A Tutorial on Support Vector Machines for Pattern Recognition," *Data Mining and Knowledge Discovery*, 2, 121-167.

Cortes, Corinna and Vladimir Vapnik (1995), "Support-Vector Networks", *Machine Learning*, 20, 273-297.

Cui, Dapeng and David J. Curry (2005), "Prediction in Marketing Using the Support Vector Machine," *Marketing Science*, 24 (4), 595-615.

Dunn, J.C. (1974), "Well-Separated Clusters And Optimal Fuzzy Partitions," *Journal of Cybernetics*, 4 (1), 95-104.

Eliashberg, Jehoshua, Sam K. Hui, and Z. John Zhang (2007), "From Story Line to Box Office: A New Approach for Green-Lighting Movie Scripts," *Management Science*, 53 (6), 881-893.

Feldman, Ronen and James Sanger (2007), *The Text Mining Handbook: Advanced Approaches in Analyzing Unstructured Data*. New York: Cambridge University Press.

Gamon, Michael, Anthony Aue, Simon Corston-Oliver, and Eric Ringger (2005), "Pulse: Mining Customer Opinions from Free Text," in *Advances in Intelligent Data Analysis VI*: Springer.

Hastie, Trevor, Robert Tibshirani, and Jerome Friedman (2009), *The Elements of Statistical Learning: Data Mining, Inference, and Prediction: Data Mining, Inference, and Prediction*, 2nd ed. New York, NY: Springer.

Ho, Tin Kam (1995), "Random Decision Forests," *Proceedings of the 3rd International Conference on Document Analysis and Recognition*, Montreal, QC, 14–16 August 1995, 278–282.

Joachims, Thorsten (1998), "Text Categorization with Support Vector Machines: Learning with Many Relevant Features," *Machine learning: ECML-98*, 137-142.

John, George H. and Pat Langley (1995), "Estimating Continuous Distributions in Bayesian Classifiers," *Proceedings of the Eleventh Conference on Uncertainty in Artificial Intelligence*, 338-345.

Malouf, Robert (2002), "A Comparison of Algorithms for Maximum Entropy Parameter Estimation," *Proceedings of the Sixth Conference on Natural Language Learning*, 20, 49-55.

Marcus, Mitchell P., Mary Ann Marcinkiewicz, and Beatrice Santorini (1993), "Building A Large Annotated Corpus of English: The Penn Treebank," *Computational Linguistics*, 19 (2), 313-330.

Meena, Arun and T.V. Prabhakar (2007), "Sentence Level Sentiment Analysis in the Presence of Conjuncts Using Linguistic Analysis," in *Advances in Information Retrieval*, Giambattista Amati and Claudio Carpineto and Giovanni Romano, eds. Vol. 4425: Springer Berlin Heidelberg.

Pedregosa, Fabian, Gaël Varoquaux, Alexandra Gramfort, Vincent Michel, Bertrand Thirion, Olivier Grisel, Mathieu Blondel, Peter Prettenhofer, Ron Weiss, Vincent Dubourg, Jake Vanderplas, Alexandre Passos, David Cournapeau, Matthieu Brucher, Matthieu Perrot, Édouard Duchesnay (2011), "Scikit-Learn: Machine Learning in Python," *The Journal of Machine Learning Research*, 12 (85), 2825-2830.

Punj, Girish and David W. Stewart (1983), "Cluster Analysis in Marketing Research: Review And Suggestions for Application," *Journal of Marketing Research*, 20 (2), 134-148.

Řehůřek, Radim and Petr Sojka (2010), "Software Framework for Topic Modelling with Large Corporam," *Proceedings of the LREC 2010 Workshop on New Challenges for NLP Frameworks*.

Rousseeuw, Peter J. (1987), “Silhouettes: A Graphical Aid to The Interpretation And Validation of Cluster Analysis,” *Journal of Computational and Applied Mathematics*, 20 (Nov), 53-65.

Rifkin, Ryan and Aldebaro Klautau (2004), “In Defense of One-Vs-All Classification,” *Journal of Machine Learning Research*, 5, 101-141.

Täckström, Oscar and Ryan McDonald (2011), "Semi-Supervised Latent Variable Models for Sentence-Level Sentiment Analysis," *Proceedings of the 49th Annual Meeting of the Association for Computational Linguistics: Human Language Technologies: short papers-Volume 2*, 569-574.

Ward Jr., Joe H. (1963), “Hierarchical Grouping to Optimize An Objective Function,” *Journal of the American Statistical Association*, 58 (301), 236–244.

Wei, Chih-Ping, Yen-Ming Chen, Chin-Sheng Yang, and Christopher C. Yang (2009), "Understanding What Concerns Consumers: A Semantic Approach to Product Feature Extraction from Consumer Reviews," *Information Systems and e-Business Management*, 8 (2), 149-167.

Yi, Jeonghee, Tetsuya Nasukawa, Razvan Bunescu, and Wayne Niblack (2003), "Sentiment Analyzer: Extracting Sentiments about A Given Topic Using Natural Language Processing Techniques," *Proceedings of the Third IEEE International Conference on Data Mining*, 427-434.
